# Dapagliflozin-intermittent fasting combination maximizes weight and metabolic regulation through AMPK/sirtuins/clock genes and gut microbiota signaling in high-fat diet-induced obesity: a novel anti-obesity approach

**DOI:** 10.1186/s13578-026-01557-4

**Published:** 2026-04-12

**Authors:** Mahmoud A. Senousy, Rana Mohamed Abo-Elmaaty, Nesreen Nabil Omar, Hanan M. Abdelgawad

**Affiliations:** 1 Department of Biochemistry, Faculty of Pharmacy, Cairo University, Cairo, 11562 Egypt; 2 Department of Biochemistry, Faculty of Pharmacy and Drug Technology, Egyptian Chinese University, Cairo, 11786 Egypt; 3 Department of Biochemistry, Faculty of Pharmacy, Modern University for Technology and Information (MTI), Cairo, Egypt

**Keywords:** BMAL1, CLOCK, GPR43, Microbiota, Short-chain fatty acids, Weight loss

## Abstract

Innovative and affordable treatment options are required to combat obesity and its detrimental impact on health economics. Dapagliflozin (Dapa), an antidiabetic medication, promotes weight loss; however, the extent of weight reduction may be limited. Intermittent fasting (IF) is a dietary approach regarded as a cost-effective and readily accessible regimen. This study investigated the beneficial effects of Dapa, IF, and their combination on weight loss and metabolic derangements in high-fat diet (HFD)-induced obesity in rats. Male Sprague-Dawley rats were allocated into 6 groups: groups 1 (normal control) and 2 (drug control) were administered a normal chow diet, while the obesity groups 3, 4, 5, and 6 were subjected to HFD for 8 weeks. Rats in groups 4, 5, and 6 underwent a further 8-week treatment with Dapa (5 mg/kg, p.o) daily, IF (16/8), or a combination of both, respectively. The HFD group exhibited elevated anthropometric measurements and adiposity index. Upon histopathological examination, the HFD group showed adipocyte hypertrophy, hypercellularity, and possible necrosis, along with hepatic fat accumulation and elevated serum liver enzymes. The HFD group showed a downregulation of p-AMPK/SIRT1 and an upregulation of SIRT7, GPR43, and clock genes BMAL1, CLOCK, and CRY1 expression in adipose tissue, along with a drop in the gut microbiome diversity, serum short-chain fatty acids (SCFAs), POMC, and PYY levels. Dapa and IF combination demonstrated favorable outcomes over monotherapy, as evidenced by normalized anthropometric measurements, improved histopathological and biochemical derangements, regulated p-AMPK/SIRT1, SIRT7, and clock genes expression, and restored gut microbiome and SCFA levels. Conclusively, this study suggests the concurrent administration of Dapa and IF as a new, beneficial, cost-effective anti-obesity strategy.

**Supplementary Information:**

The online version contains supplementary material available at 10.1186/s13578-026-01557-4.

## Introduction

Obesity is a multifaceted, chronic, and progressive trait accompanied by diminished quality of life, elevated mortality, and shortened life expectancy [1]. It is a major global health issue, reaching epidemic proportions. Obesity has reached a substantial global prevalence, with 43% of adults being overweight and 16% having obesity in 2022 [2]. It is estimated that 51% of people will be overweight or obese by 2035 [3]. Obesity is not merely a comorbidity but a major etiological factor in the development of type 2 diabetes and heart failure [4], posing a significant obstacle to sustainable development.

Energy homeostasis and body weight regulation are influenced by dynamic interactions among environmental, genetic, and epigenetic factors, which also impact the pathogenesis of obesity [5]. Dietary fat consumption is a major environmental contributor to increased adiposity [6]. Adipose tissue functions as a crucial endocrine organ for regulating systemic energy metabolism. It has three main categories: white adipose tissue (WAT) for storing energy, brown adipose tissue (BAT) for utilizing energy for thermoregulation, and beige adipose tissue, a hybrid type that exhibits features of both WAT and BAT [7]. Targeting BAT activation and WAT browning offers therapeutic potential for combating the obesity epidemic, given their beneficial effects on energy balance [8]. Furthermore, additional treatment regimens for mitigating fat accumulation in the adipose tissue are necessary to tackle this epidemic [7, 8].

Among the genetic contributors to obesity are sirtuins (SIRTs) [9]. They constitute a class of NAD^+^-dependent protein deacetylases, with certain members exhibiting established functions in energy metabolism [9]. SIRT1 is activated in nutrient-depletion conditions via adenosine monophosphate-activated protein kinase (AMPK), which functions as a vital cytoplasmic energy detector [10]. SIRT7, the last mammalian sirtuin currently identified, is implicated in various aspects of aging, metabolic stress, and obesity [11, 12]. Indeed, SIRT1 inhibits adipogenesis and increases free fatty acid mobilization in the visceral WAT [13], whereas SIRT7 stimulates adipogenesis [11, 14].

Recent studies indicate a mutual regulation between the circadian clock and sirtuins, which plays roles in various signal transduction and metabolic processes [15]. The circadian clock is an intrinsic timekeeping system that regulates physiological, metabolic, and behavioral rhythms over 24 h [15]. Circadian rhythm expression is governed by clock genes, which include at least nine proteins: circadian locomotor output cycles kaput (CLOCK), brain and muscle ARNT-like protein 1 (BMAL1), reverse erythroblastosis virus α (REV-ERBα), cryptochrome (CRY1, CRY2), retinoid-related orphan receptor alpha (RORα), and period (PER1, PER2, PER3) [16]. Since rhythm desynchronization might promote the development of obesity and other metabolic diseases, there is compelling evidence supporting a bidirectional relationship between obesity and circadian rhythm disturbance [17].

Lifestyle, pharmacologic, and surgical interventions help manage obesity. Current approaches for morbid obesity mainly emphasize bariatric procedures as a long-term option for severe cases [18]. Because these have multiple negative consequences [18], there is a strong interest in finding safe and cost-effective anti-obesity regimens for weight reduction amid the global obesity epidemic.

Dapagliflozin (Dapa) is an antidiabetic agent that reduces renal glucose reabsorption by blocking the sodium-glucose co-transporter 2 (SGLT2) [19]. Additionally, it has antioxidant and anti-inflammatory effects [19]. Dapa positively influences weight loss; however, body weight reduction may be limited by hyperphagia. This effect potentially results from a compensatory response to the negative energy balance caused by uncontrolled glucose excretion or a nutrient-deprived state induced by Dapa [20–22]. This hyperphagia may be associated with gut microbiome dysbiosis, as noted in diabetic rats treated with Dapa [23].

The gut microbiota, a complex and interconnected system, is considered a core organ due to its diverse interactions with other organs via neurological, hormonal, and metabolic pathways [24]. This microbial ecosystem is primarily composed of two phyla: Firmicutes, which encompasses genera such as Lactobacillus, Faecalibacterium, Enterococcus, and Clostridium, and Bacteroidetes, which includes notable genera like Prevotella and Bacteroides [25]. Other phyla, such as Verrucomicrobia, Actinobacteria, Proteobacteria, and Euryarchaeota, are present in lower concentrations [25]. One of the gut microbiome’s key functions is the degradation of dietary soluble fibers through fermentation and anaerobic processes, producing short-chain fatty acids (SCFAs) [24]. The primary SCFAs are acetate, propionate, and butyrate. They account for roughly 80% of total SCFAs and perform various essential functions in the human body [26]. These SCFAs diminish energy intake, body weight, and adiposity, and modify hypothalamic neuronal activation dynamics. Indeed, SCFAs modulate appetite-regulating peptides via binding to free fatty acid receptors, including G-protein-coupled receptor 41 (GPR41), GPR109a, and GPR43 [26, 27]. However, Dapa-mediated alterations in the gut microbiota in obesity and its mechanistic relationship to appetite regulation and weight loss need further investigation.

Intermittent fasting (IF) is a nutritional approach characterized by alternating patterns of fasting and eating at fixed intervals. It encompasses three primary types: time-restricted eating, alternate-day fasting (ADF), and twice-per-week fasting [28]. Time-restricted eating typically employs a restricted eating period within 24 h, mainly the 16:8 model [28]. ADF represents eating days interspersed with fasting days, allowing only water consumption on fasting days, referred to as zero-calorie ADF. Twice-per-week fasting, an updated ADF version, involves two fasting days per week, which may be consecutive or non-consecutive [29]. IF restricts food intake to designated periods while preserving the type and quantity of food ingested during non-fasting intervals. This method promotes sustained adherence to the benefits of fasting, unlike extended caloric restriction that frequently leads to low patient compliance [29]. IF influences the synchronization of metabolic and behavioral circadian clock genes, potentially offering benefits in animal studies [30]. IF may also alter the gut microbiome [31]. Thereby, it can be hypothesized that IF may be a promising strategy to address the Dapa-induced hyperphagia.

In this context, this study assessed the potential impact of Dapa, IF, and their combination on weight loss and metabolic derangement in high-fat diet (HFD)-induced obesity in rats, with an emphasis on sirtuins, clock genes, appetite-regulating peptides, and gut microbiome/SCFAs/GPR43 receptor signaling.

## Materials and methods

### Drugs and chemicals

Dapa was procured as Forxiga® (AstraZeneca AB, Södertälje, Sweden). Carboxymethyl cellulose (CMC) was supplied from El-Nasr Chemical Co (Cairo, Egypt). Components of HFD such as soybean oil and choline chloride were procured from ABCHEM (Mansoura, Egypt). Other HFD ingredients were purchased from a commercial source of the highest grade.

### Animals

Six-week-old Sprague–Dawley rats (150–170 g) were used in the experimental procedures. Animals were purchased from the laboratory animal research center, Faculty of Medicine, Ain Shams University. The rats were kept in standard polypropylene cages (5 per cage) under standard conditions: 12/12-h light/dark cycles, with lights on at 6:00 a.m. (zeitgeber time 0), humidity (60% ± 10%), and constant temperature (22 ± 2°C). The rats were fed a normal rodent chow diet (EL Nasr Pharmaceutical Chemicals Co., Cairo, Egypt), allowed ad libitum, with unrestricted access to water and acclimatized to the environment for two weeks before the experiment.

The Institutional Review Board of the Faculty of Pharmacy, Cairo University, Egypt gave its clearance to the study (approval code: BC3058). The research ethics committee at the Faculty of Medicine, Ain Shams University (FMASU-REC) also approved the work. The FMASU-REC general guidelines were followed when administering animal procedures and care, and they adhered to the ARRIVE guidelines’ principles, the U.K. Animals (Scientific Procedures) Act, 1986, and related guidelines; EU Directive 2010/63/EU for animal experiments, where suitable precautions were taken to prevent the rats from experiencing pain and suffering.

### Experimental design and obesity induction

**Fig. 1:**
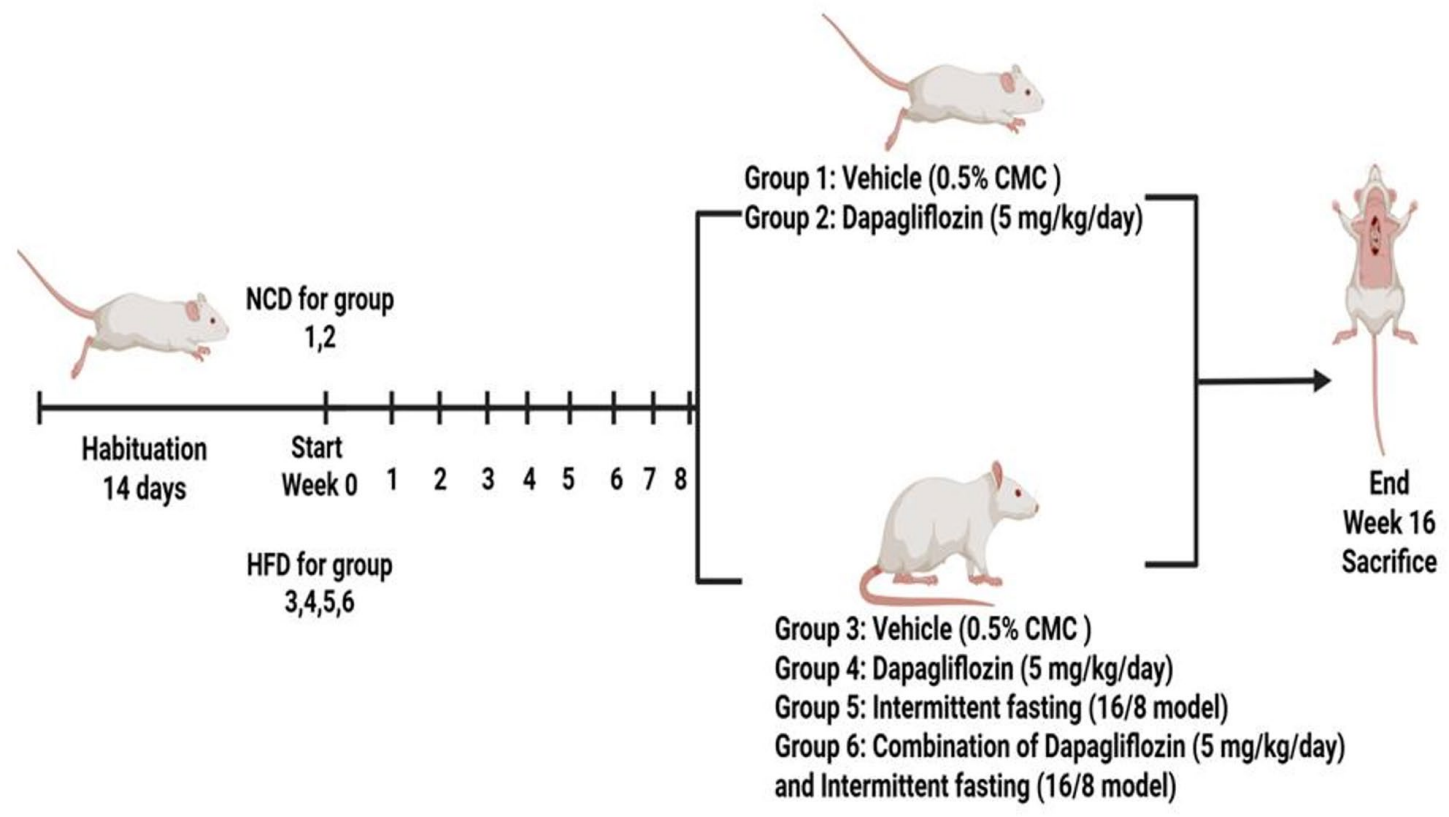
Experimental design and timeline of the study. CMC, carboxymethyl cellulose; HFD, high-fat diet; NCD, normal chow diet

Sixty rats were included at the study beginning and allocated into six groups (10 rats/group) at random. Two rats from the normal control group died during the early phase of the experiment at weeks 2 and 3, respectively, resulting in a final sample size of eight rats in this group. No animal loss occurred in the other experimental groups. The experimental study was designed to be 16 weeks long, including two distinct phases: an obesity induction phase (week 0–8) using HFD, followed by an intervention phase (week 8–16) concurrently with HFD. Obesity was confirmed at week 8, which was, therefore, defined as the baseline for treatment evaluation (Fig. 1).

Administration of HFD for 8 weeks was used to induce obesity with the following ingredient composition: 35.8 g soybean oil, 23.5 g casein, 2.7 g sucrose, 15 g maize starch, 17.9 g fiber, 1 g vitamin mix, 3.5 g mineral mix, 0.3 g L-cysteine, and 0.25 g choline chloride per 100 g [32]. In this diet, the total calories from fats counted for 65%.

The experimental design was created as stated below:

Group 1 (normal control group): Rats were given a normal chow diet for 8 weeks, then they were given 0.5% CMC (vehicle of Dapa) via oral gavage for an additional 8 weeks.

Group 2 (Dapa-control group, Dapa): Rats were given a normal chow diet for 8 weeks, and then they received Dapa (5 mg/kg/day) [33] in 0.5% CMC via oral gavage for an additional 8 weeks.

Group 3 (HFD-induced obesity group, HFD): Rats were given HFD for 8 weeks [32], and then the obese rats were given 0.5% CMC via oral gavage for an additional 8 weeks.

Group 4 (Dapa-treated group, HFD/Dapa): Rats were given HFD for 8 weeks [32], then the obese rats received Dapa (5 mg/kg/day) [33] dissolved in 0.5% CMC via oral gavage for an additional 8 weeks.

Group 5 (IF-treated group, HFD/IF): Rats were given HFD for 8 weeks [32], then the obese rats were allowed to fast for 16 h/day for an additional 8 weeks. HFD was available to rats only for 8 h from 8:00 am to 4:00 pm.

Group 6 (Dapa and IF combination group, HFD/Dapa + IF): Rats were given HFD for 8 weeks [32], then the obese rats received Dapa (5 mg/kg/day) [33] dissolved in 0.5% CMC via oral gavage and concurrently were allowed to fast for 16 h/day for an additional 8 weeks. HFD was available to rats only for 8 h from 8:00 a.m. to 4:00 p.m.

The dose of Dapa was chosen as outlined in a previous study by Devenny et al. in a rat model of diet-induced obesity [33], where daily doses of 0.5 to 5 mg/kg of Dapa were administered to rats for 38 days. According to this prior study, the largest weight reduction was obtained at the highest dose (5 mg/kg/day) compared with vehicle-treated controls [33].

The following formula can be used to calculate the human equivalent dose (HED) (mg/kg): Animal dose (mg/kg) × (Animal K_m_ /Human K_m_). The ratio of a species’ mean body weight (kg) to its body surface area (m^2^) yields the correction factor (K_m_), which stays constant at 37 for humans and 6 for rats.

### Anthropometric measurements

At the study beginning, baseline assessments of body weight (BW) and anthropometric parameters of the rats were conducted as previously described. The measured parameters included abdominal circumference (AC), taken nearest to the hind foot at the largest part of the rat abdomen [34]; thoracic circumference (TC), measured just behind the foreleg [35]; body length, defined as the distance from nose to anus [35]; and body mass index (BMI) calculated as body weight (g)/body length^2^ (cm^2^) [35]. The measurements were made in anesthetized rats using 0.1 mL of 1% sodium barbiturate intraperitoneally [35]. The anthropometric measurements were repeated after the first 8 weeks to ascertain the obesity status. Finally, the anthropometric measurements were further assessed upon completion of the study.

### Estimation of food and energy intakes

Daily food intake was monitored throughout the experimental period. For inter-group comparison, food and energy intakes were estimated and reported throughout the 8-week treatment period (intervention phase, i.e., 56 days). The amount of diet provided to each group was initially weighed, and the remaining food was weighed after 8 h. Food intake per rat was estimated as the difference between food offered and food remaining after 8 h divided by the number of rats per group, and expressed as g/rat/8 h. Energy intake (expressed as kcal/rat/8 h) was estimated by multiplying the food intake by the diet-specific energy density (kcal/g), according to the manufacturer’s specifications.

### Blood collection, tissue processing, and calculation of adiposity and liver indices

Upon completion of the experimental period, blood samples were obtained from the retro-orbital plexus of all rats, following an overnight fasting period of 12–14 h. For serum separation, the samples were placed in plain tubes, kept to clot for 15 min at room temperature, and subsequently centrifuged at 600×*g* for 10 min. The serum was stored at –80 °C after fractionation into four aliquots and then used for biochemical measurements.

Rats were anesthetized using 3% isoflurane inhalation. Animals were sacrificed by rapid decapitation at 8:00 a.m. (zeitgeber time 2), ensuring that tissue collection occurred at the same circadian time across all experimental groups. Tissue sampling was completed within a narrow time window to minimize circadian variability. To standardize metabolic conditions, all animals underwent overnight fasting prior to blood collection and subsequent sacrifice. Accordingly, all groups were sampled in a comparable fasting state, thereby eliminating potential confounding effects of feeding status on clock gene expression. The adipose tissue from the epididymis, retroperitoneal, and inguinal fat layers of each rat was collected, washed with phosphate-buffered saline (PBS), dried, and weighed. The adiposity index (%) was calculated using the following equation: epididymis fat + retroperitoneal fat + inguinal fat weight × 100 / final body mass. The liver was also collected, rinsed with PBS, dried, and weighed. The liver index (%) was calculated to indicate the liver size relative to the overall body mass using the equation: liver weight / final body weight × 100.

The adipose tissue from each rat was then divided into 3 portions. One portion was fixed in 10% (v/v) buffered formalin for 24 h and processed for routine histology staining. The other two portions were kept frozen at –80°C until use in western immunoblotting and real-time PCR. The liver was fixed in 10% (v/v) buffered formalin for 24 h for histopathological examination. In addition, specimens of the large intestine contents (whole stool) were collected from each rat in sterile stool collection containers and stored at –80°C until used in gut microbiome analysis.

### Histopathological examination

Adipose and liver tissue samples, of three rats in each group, preserved in 10% buffered formalin, were subsequently inserted in paraffin blocks, and serial sections with a 5-µm thickness were prepared. To examine the general morphology of the adipose tissue and liver, hematoxylin and eosin (H & E) staining was applied to the obtained segments. All slides were evaluated by a qualified histopathologist using a light microscope in a blinded manner. The micrographs were taken by a Full HD microscopic camera supplied by Leica Microsystems GmbH, Wechsler, Germany. For each sample, at least six representative, non-overlapping fields per tissue section were randomly selected and examined.

### Biochemical assays

#### Colorimetric assays

A diagnostic glucose assay kit provided by Sigma Aldrich (Saint Louis, USA, product code GAGO-20) was used to measure serum glucose levels. The serum lipid profile, which included total cholesterol, triglycerides, and HDL-cholesterol (HDL-C) levels, was determined employing diagnostic assay kits supplied by Abcam (Cambridge, UK, product code ab102515), Cayman Chemical (Michigan, USA, product code 10010303), and Biochain (Hayward, USA, product code Z5030057), respectively. Serum LDL-C level was estimated based on the Friedewald equation. Serum alanine aminotransferase (ALT) and aspartate aminotransferase (AST) activities were determined using diagnostic assay kits supplied by Spectrum Diagnostic (Egypt, Cairo, product code 264 002 and 260 002, respectively).

#### Enzyme-linked immunosorbent assay (ELISA)

Serum insulin, leptin, and proopiomelanocortin (POMC) levels were assessed utilizing rat ELISA kits supplied by MyBioSource (California, USA, Catalog No: MBS281388, MBS761173, and MBS2023251, respectively). A rat ELISA kit provided by BioVision (Milpitas, USA, Catalog No: K4903-100) was employed to assess serum adiponectin levels. Serum peptide YY (PYY) levels were evaluated using a rat ELISA kit supplied by Elabscience (Wuhan, China, Catalog No: E-EL-R0720), following the vendor’s instructions.

#### Reverse transcriptase-quantitative polymerase chain reaction (RT-qPCR)

RT-qPCR was employed for the assay of SIRT1, SIRT7, GPR43, and clock genes (BMAL1, CLOCK, and CRY1) mRNA expression levels in the rat adipose tissue. Total RNA was isolated from adipose tissue lysate using direct-zol RNA miniprep plus provided by Zymo Research (California, USA). In brief, a part of adipose tissue was lysed by grinding with pestle and mortar. The grounded tissue was then added to a TRI reagent, bound directly to the Zymo-spin column, and then washed. RNA was eluted in RNase-free water for immediate application in downstream processes. The RNA quantity and purity were assessed using a Beckman dual spectrophotometer (California, USA), with applying the vendor’s protocol.

Reverse transcription and real-time PCR were conducted in a single step with the SuperScript IV One-Step RT-PCR kit (Thermo Fisher Scientific, Waltham, MA, USA) using the StepOne Real-Time PCR System (Applied Biosystems, Foster City, CA, USA), adhering to the manufacturer's protocol. GAPDH was set as the reference gene. Target gene primers were designed by the NCBI Primer-BLAST tool, and subsequently custom manufactured by Invitrogen (Waltham, USA). The primer sequences are listed in Table 1.

**Table 1:** Primer sequences used in qPCR

Gene	Forward primer (5’-3’)	Reverse primer (5’-3’)
SIRT1	CTATACCCAGAACATAGACACG	ACAAATCAGGCAAGATGC
SIRT7	GCAGAGCAGACACCATCC	GTTCACGATGTAAAGCTTCG
GPR43	ACCATCGTCATCATCGTTCA	ACGAAGCGCCAATAACAGAA
BMAL1	TGGCCAGAGTGAATGCTTTTG	CCTGACTGGCCTGGAACTTG
CLOCK	TGCTGTCCTTACTGCTTGGT	TGGCAAAGTGGTGATACCTGA
CRY1	TGCGCATTTCACACACACTG	GACAGAGGGGTTGTGCACTT
GAPDH	TGGATTTGGACGCATTGGTC	TTTGCACTGGTACGTGTTGAT

A negative control was used in each PCR run to assure the absence of contamination. Melting curve analysis was implemented to verify the PCR products' specificity and exclude the possibility of primer dimers or hairpin structures. Following each PCR run, the data were represented in terms of cycle threshold (Ct). Each target gene's relative quantification was calculated and normalized to the housekeeping gene using the delta-delta Ct (ΔΔCt) method. The 2^−∆∆Ct^ formula was utilized to present the fold change.

#### Western blotting

Western blotting was employed to evaluate phosphorylated AMPK (p-AMPK) protein levels in the rat adipose tissue. Proteins were isolated from adipose tissue and purified utilizing a mirVana PARIS kit (California, USA), complying with the manufacturer’s instructions. Protein fraction concentration was determined by applying the Bradford method [36]. Gel electrophoresis was performed using a western blot system kit provided by Advansta (California, USA). Following gel polymerization, 30 µg of proteins were loaded on SDS-PAGE, electrophoresed, and then transferred to a polyvinylidene difluoride membrane. The membrane was incubated in a blocking buffer (5% non-fat dried milk in 10 mM Tris–HCl, pH 7.5, 0.1% Tween 20, and 100 mM NaCl) for 1 h at room temperature. After that, the membrane was incubated overnight at 4°C with primary antibodies against p-AMPK (Phospho-AMPKα Thr172 Antibody, abcam 2531) and β-actin (housekeeping protein) (Anti-β-actin antibody, abcam 8224), with a 1:1000 dilution ratio. The membrane was washed using a washing buffer (25 mM Tris, pH 7.4, 0.15 M NaCl, and 0.1% Tween 20) at room temperature for 30–60 min and then incubated for 1 h at room temperature with HRP-conjugated secondary antibody (Proteintech, SA00001-0), with a 1:3000 dilution ratio. Totallab analysis software v1.0.1 (www.totallab.com) was used along with the gel documentation system (Geldoc-it, UVP, England) for data analysis.

### Next-generation 16S rRNA sequencing method for the determination of gut microbiome

DNA extraction from the rat intestinal contents (250 mg) was performed employing the QIAamp PowerFeca Pro DNA Kit (Hilden, Germany), based on the vendor’s recommendations. The next-generation 16S rRNA sequencing method was conducted using the Illumina MiSeq 16S metagenomic sequencing kit (California, USA) for gut microbiome determination. Briefly, DNA was amplified using the PCR technique employing primers that target the hypervariable regions, such as the V3 and V4 regions of the 16S rRNA gene. The sequences of the Illumina overhang adapters were incorporated to the locus-specific primers for the targeted region, as listed in Table 2. For amplification, the PCR master mix was prepared by adding 2.5 µL microbial DNA (5 ng/µL), 5 µL amplicon PCR forward primer (1 µM), 5 µL amplicon PCR reverse primer (1 µM), and 12.5 µL KAPA HiFi HotStart readymix (1x) to reach a final volume of 25 µL. PCR was performed using a preheated thermal cycler (California, USA) under the specified thermal conditions: initial denaturation at 95 °C for 3 min, followed by amplification through 25 cycles. Each cycle includes denaturation at 95 °C for 30 s, annealing at 55 °C for 30 s, and extension at 72 °C for 30 s. A final extension is performed at 72 °C for 5 min.

**Table 2:** The Illumina overhang adapter sequences and full-length primer sequences used

Forward overhang: 5’TCGTCGGCAGCGTCAGATGTGTATAAGAGACAG3’ [locus-specific sequence]
Reverse overhang: 5’GTCTCGTGGGCTCGGAGATGTGTATAAGAGACAG3’ [locus-specific sequence]
16S Amplicon PCR Reverse Primer 5'GTCTCGTGGGCTCGGAGATGTGTATAAGAGACAGGACTACHVGGGTATCTAATCC
16S Amplicon PCR Forward Primer 5'TCGTCGGCAGCGTCAGATGTGTATAAGAGACAGCCTACGGGNGGCWGCAG

To prepare a microbiome library, the PCR products were fragmented to create uniform-sized DNA fragments by adding AMPure XP beads, 80% ethanol, 10 mM Tris pH 8.5, then centrifuged at 6000×*g* at 20 °C for 3 min. Illumine specific adapters and specific index sequences were added and ligated to the end of DNA fragments employing the Nextera XT index kit (California, USA). PCR was repeated to enrich the quantity of the library used in the sequencing step. For purification, 80% ethanol and 10 mM Tris pH 8.5 were added to the library, and then centrifuged at 1000×*g* at 20 °C for 1 min. The library was quantified using PicoGreen Illumine fluorometric quantification kit.

On the Illumine sequencing platform, the library was loaded, then clustered onto a flow cell, with each cluster illustrating a specific DNA fragment. The sequence of the DNA was read from each cluster using the Illumina MiSeq Reporter software (MSR). The metagenomics workflow was picked to classify organism passed on V3 and V4 amplicon using a 16S rRNA database. The data sequenced was analyzed and processed to assemble sequences, and then the Greengenes database (http://greengenes.lbl.gov/) and the premium curated Micro SEQ ID 16S rRNA reference database were used for selecting the operational taxonomic unit (OTU).

### Gas chromatography-mass spectrometry (GC–MS) for the determination of SCFAs

The quantitative analysis of serum SCFAs was performed using GC–MS on a TRACE GC ultra-gas chromatograph from Thermo Scientific Corp. (USA), in conjunction with a thermo mass spectrometer detector (ISQ™ EC Single Quadrupole Mass Spectrometer). Serum SCFAs were extracted utilizing the Folch method, with a chloroform:methanol ratio of 2:1 (v/v) [37]. Samples were diluted with hexane at a 1:10 ratio, and subsequently 1 μL of the mixture was injected. An internal standard solution consisting of 2-ethylbutyric acid at a concentration of 1 mg/L, prepared in demineralized water, was used. The GC–MS analysis utilized helium as the carrier gas at a flow rate of 1.0 mL/min and a split ratio of 1:10, following the specified temperature program: initial temperature of 60ºC for 1 min, followed by a ramp rate of 4ºC/min to a final temperature of 240ºC, maintained for 1 min. The TR-5 MS column with a length of 30 m, a diameter of 0.32 mm, and a thickness of 0.25 μM was utilized. The injector and detector were maintained at 210ºC. Mass spectra were obtained through electron ionization at 70 eV, utilizing a spectral range of mass-to-charge ratio (m/z) from 40 to 450.

 By examining the peak areas of the corresponding acids, the concentrations of serum SCFA were measured. Using their retention indices, mass spectra compared to real standard, and reference data from the Wiley spectral library collection, the SCFA components were deconvoluted and identified using the AMDIS software (www.amdis.net).

The limits of detection (LOD) for acetate, propionate, and butyrate were 0.18 mg/L, 0.22 mg/L, and 0.14 mg/L, respectively, while the limits of quantification (LOQ) for acetate, propionate, and butyrate were 0.60 mg/L, 0.75 mg/L, and 0.47 mg/L, respectively.

Following the full-scan mode, a targeted GC–MS selected ion monitoring (SIM) approach was used to reduce interference and detect low-abundance SCFAs. The same internal standard, extraction protocol, and LOD/LOQ were applied and a specific chromatogram for each SCFA was generated.

### Statistical analysis

Using the G*Power software 3.1.9.7, a power analysis was conducted to determine the group size (power = 0.8, α = 0.05). GraphPad Prism version 10.2.3 (California, USA) was used for statistical analyses. Outliers, homogeneity of variance, and the normality assumption were verified in the data. The normal distribution of the variables was tested using the Shapiro–Wilk test. To check for outliers, Dixon's Q test was employed (no outliers were detected at 95% confidence, and no data points were excluded). One-way ANOVA and Tukey–Kramer's post-hoc tests were used to evaluate the data, and the results are displayed as mean ± standard deviation (SD). Tukey's HSD post-hoc analysis produced adjusted *P*-values. ANOVA results are reported as F (DFn, DFd) and *P*-value. A *P*-value of less than 0.05 was determined to be statistically significant.

## Results

### Effects of Dapa, IF, and their combination on the anthropometric measurements in HFD-induced obesity in rats

**Fig. 2:**
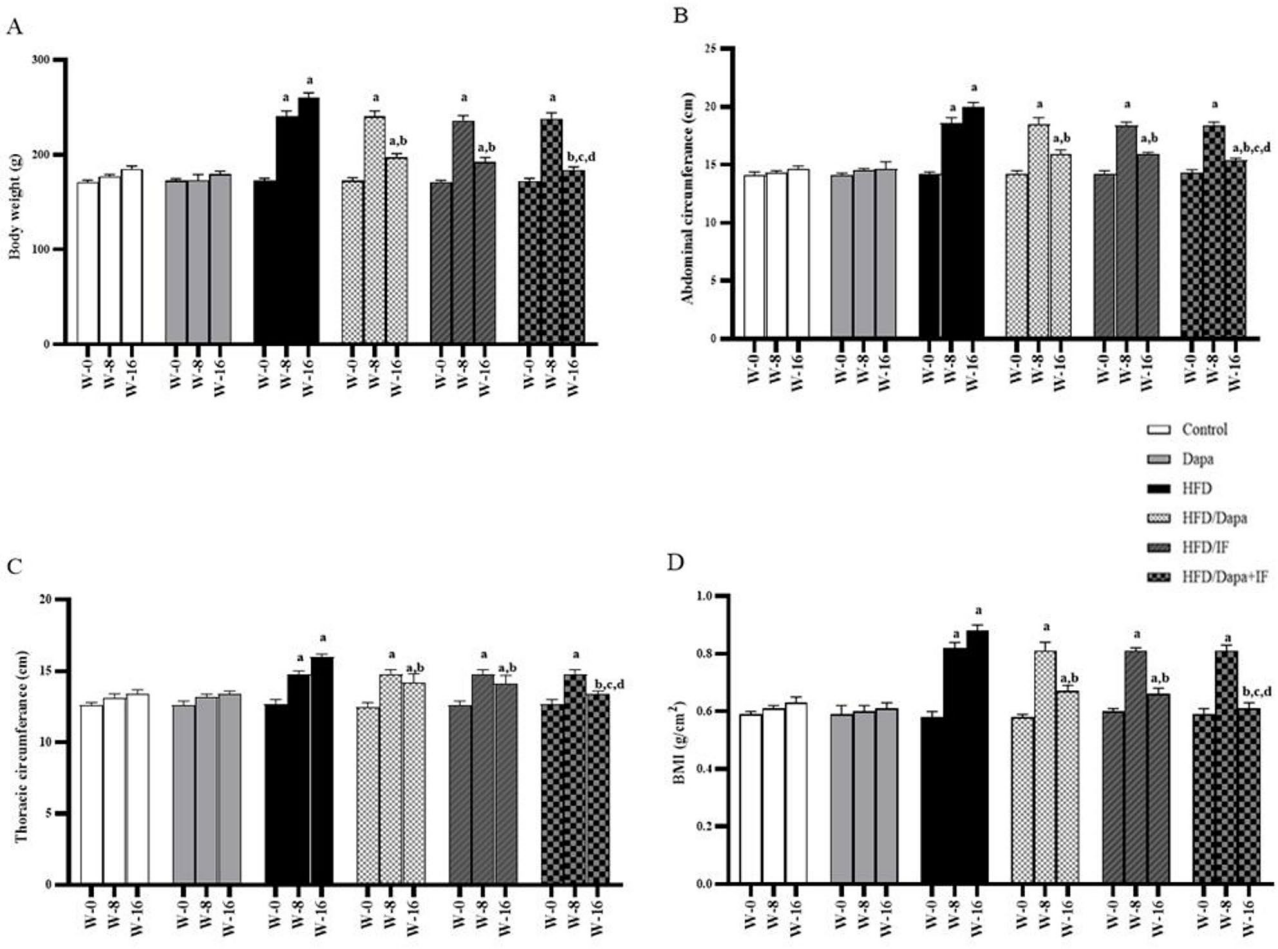
Effects of Dapa, IF, and their combination on the anthropometric measurements in HFD-induced obesity in rats. **A** Body weight at weeks 0, 8, and 16. **B** Abdominal circumference at weeks 0, 8, and 16. **C** Thoracic circumference at weeks 0, 8, and 16. **D** Body mass index (BMI) at weeks 0, 8, and 16. Data are displayed as mean ± SD, normal control group: n = 8; other groups: n = 10. Following ANOVA, Tukey’s post-hoc test was conducted. ^a^significant from the normal control group, ^b^significant from the HFD group, ^c^significant from the HFD/Dapa, ^d^significant from the HFD/IF. The significance level was set at *P* < 0.05

Anthropometric measurements were recorded 3 times during the study: at the baseline (week 0), at the middle of the study (week 8), and at the study endpoint (week 16), as shown in Fig. 2.

At week 0, there were no significant differences in BW, AC, TC, and BMI between the six studied groups (*P* > 0.05). Meanwhile, at week 8, the levels of the four measures were significantly higher in the obesity or intervention groups (HFD, HFD/Dapa, HFD/IF, and HFD/Dapa + IF) than the normal control group (Fig. 2A-D). However, the comparison of these anthropometric measurements between obesity and intervention groups showed no statistical difference.

At week 16, the HFD group showed a significant increase in BW by 41.1%, AC by 36.1%, TC by 20.1%, and BMI by 39.7% compared to the normal control group. The HFD/Dapa, HFD/IF, and HFD/Dapa + IF-treated groups exhibited a significant decrease in BW by 24.3%, 26.4%, and 29.5%; AC by 20%, 20%, and 23.5%; TC by 11.8%, 12.4%, and 16.8%; and BMI by 23.9%, 25%, and 30.7%, respectively, compared to the HFD group. Notably, the HFD/Dapa + IF-treated group showed significantly lower levels of the four anthropometric measurements compared to the other treated groups, reaching the point of normalization in BW, TC, and BMI (Fig. 2A, C, and D)**.** To note, there were no significant differences in the HFD/Dapa versus HFD/IF groups’ comparison (*P* > 0.05).

Noteworthy, there were no statistical differences in the levels of measured anthropometric parameters between the Dapa-control group and the normal control group at weeks 8 and 16 (*P* > 0.05). Additionally, these two groups were not statistically different in all the studied histopathological and biochemical investigations, thus to enhance the conciseness their comparison will not be further discussed.

**Fig. 3:**
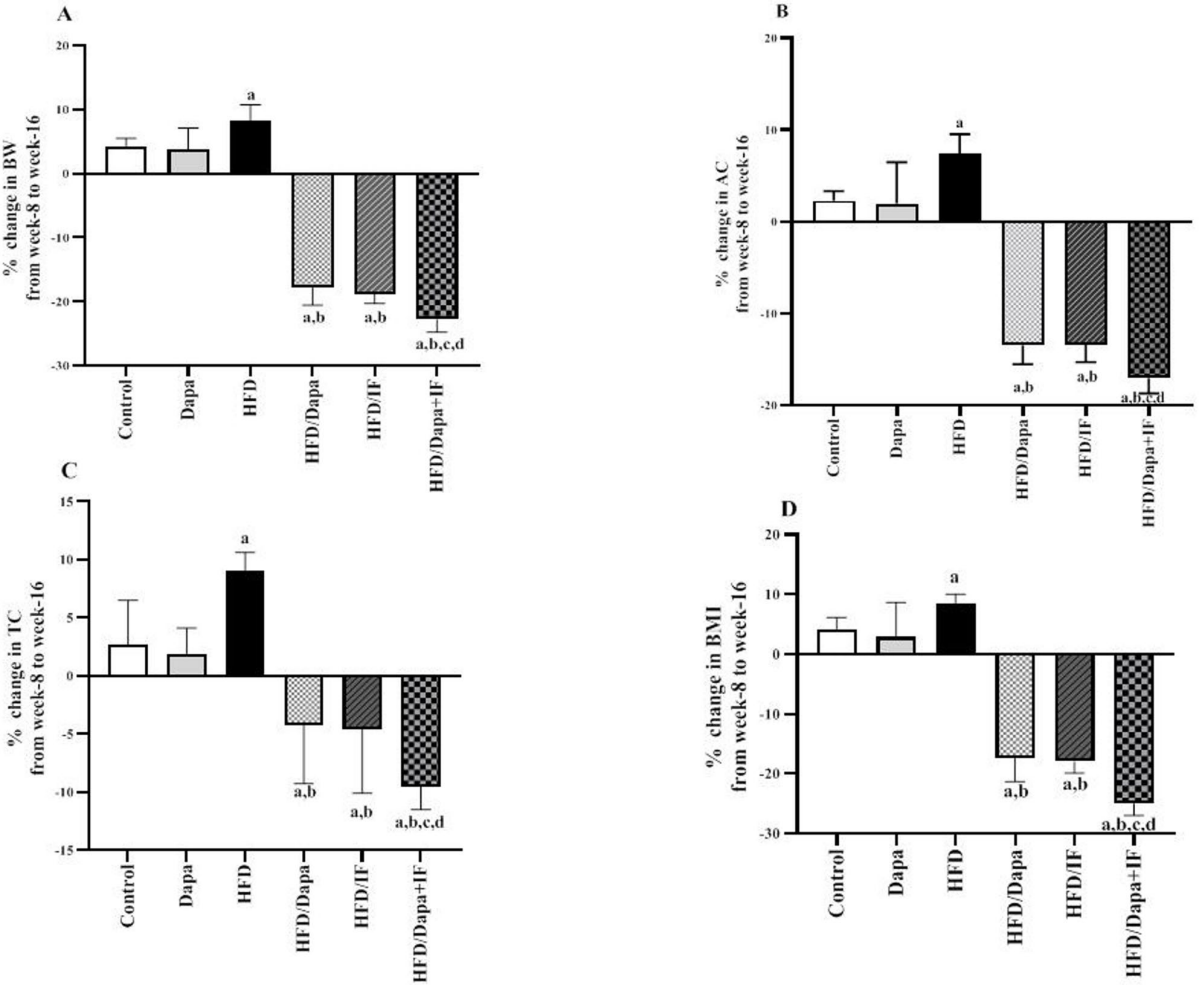
Effects of Dapa, IF, and their combination on % change in the anthropometric measurements from week 8 to week 16 in HFD-induced obesity in rats. **A** % change in body weight (BW) [F (5,52) = 351.1, *P* < 0.0001], **B** % change in abdominal circumference (AC) [F (5,52) = 166.7, *P* < 0.0001], **C** % change in thoracic circumference (TC) [F (5,52) = 31.26, *P* < 0.0001], D) % change in body mass index (BMI) [F (5,52) = 180.0, *P* < 0.0001]. Data are displayed as mean ± SD, normal control group: n = 8; other groups: n = 10. Following ANOVA, Tukey’s post-hoc test was conducted. ^a^significant from the normal control group, ^b^significant from the HFD group, ^c^significant from the HFD/Dapa, ^d^significant from the HFD/IF. The significance level was set at *P* < 0.05

After successful induction of obesity by HFD feeding for 8 weeks, week 8 was considered the baseline for treatment, and the % change in anthropometric data was calculated from this baseline to the end of the study (week 16) in each studied group. We then evaluated the effects of Dapa, IF, and their combination on the reduction of anthropometric measurements represented by % change in BW, AC, TC, and BMI from week 8 to week 16 (Fig. 3).

In this time interval, the administration of Dapa, IF, or their combination caused a significant decrease in BW by 17.9%, 18.8%, and 22.8% (Fig. 3A); AC by 13.4%, 13.4%, and 17.0% (Fig. 3B); TC by 4.2%, 4.9%, and 9.5% (Fig. 3C); and BMI by 17.3%, 17.9%, and 25.0% (Fig. 3D), respectively. Notably, the reduction of these anthropometric data was more profound in the HFD/Dapa + IF combination group compared with the HFD/Dapa and the HFD/IF-treated groups, while the single-agent groups showed no significant difference from the other.

### Effects of Dapa, IF, and their combination on food and energy intakes and adiposity index in HFD-induced obesity in rats

**Fig. 4:**
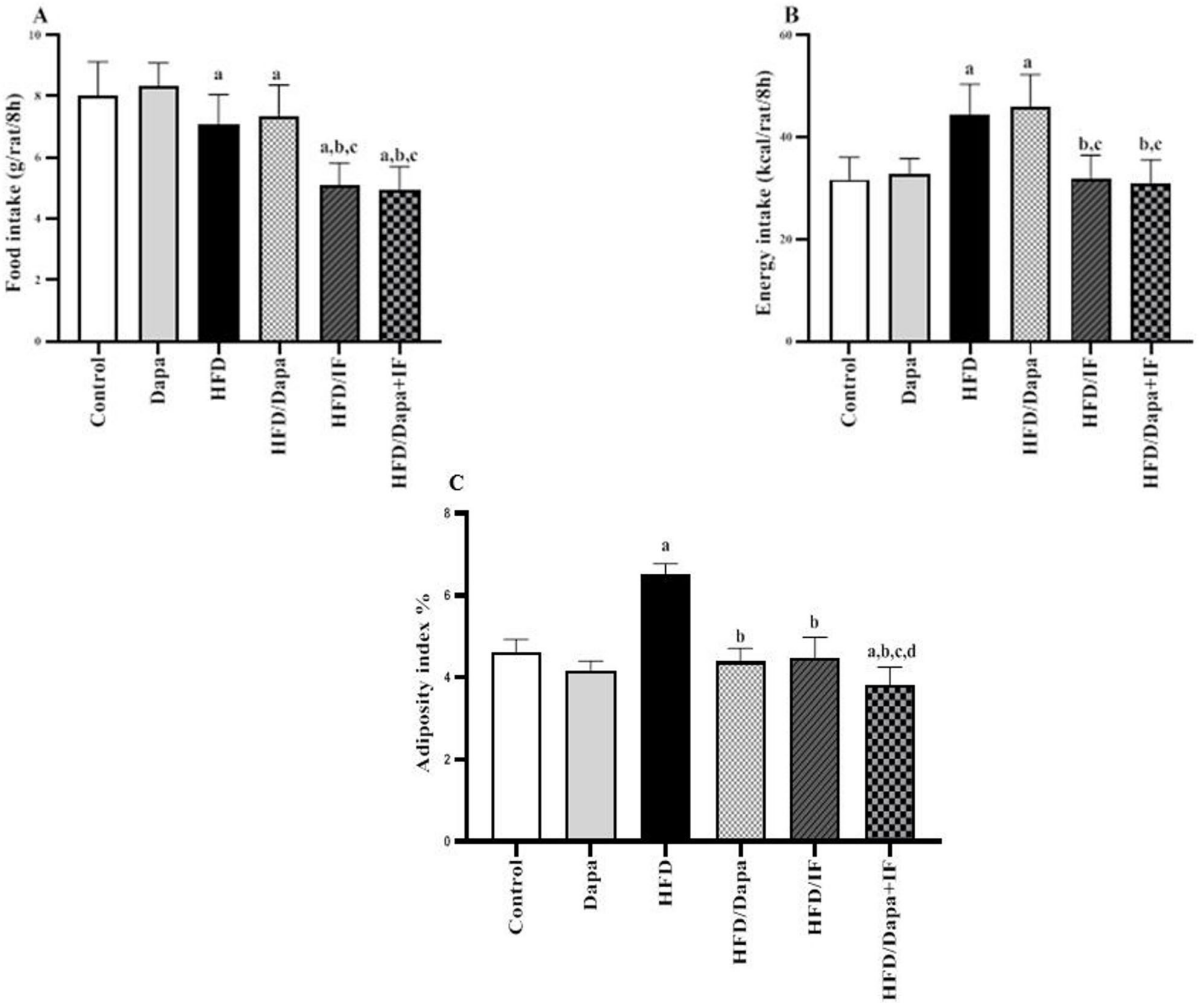
Effects of Dapa, IF, and their combination on food and energy intakes and adiposity index in HFD-induced obesity in rats. Data are displayed as mean ± SD, normal control group: n = 8; other groups: n = 10. Following ANOVA, Tukey’s post-hoc test was conducted. **A** Food intake [F (5,330) = 146.9, *P* < 0.0001]. **B** Energy intake [F (5,330) = 109.7, *P* < 0.0001]. Food and energy intakes were estimated throughout the 8-week treatment period (intervention phase, i.e., 56 days). **C** Adiposity index [F (5,52) = 68.99, *P* < 0.0001]. ^a^significant from the normal control group, ^b^significant from the HFD group, ^c^significant from the HFD/Dapa, ^d^significant from the HFD/IF. The significance level was set at *P* < 0.05

Results of food and energy intakes were reported daily during the 8-week intervention phase (56 days). As shown in Fig. 4A and B, the HFD group showed a significant decrease in the food intake by 11.8%; however, it demonstrated increased energy intake by 39.7% compared to the normal control group. Regarding intervention groups, the HFD/Dapa group showed a non-significant difference either in the food or energy intake compared to the HFD group (*P* > 0.05). The HFD/IF and HFD/Dapa + IF-treated groups exhibited a significant decrease in the food intake by 28.3% and 30.11%, respectively compared to the HFD group, while normalized the energy intake. The latter two groups showed non-significant differences in both food and energy intakes.

The adiposity index of rats from different groups was evaluated at the end of the study using adipose tissue from the epididymis, retroperitoneal, and inguinal fat layers (Fig. 4C). Administration of HFD significantly increased the adiposity index by 41.3% compared with that in the normal control group. Treatment with Dapa, IF, or their combination attenuated the effect of HFD, as indicated by decreasing the adiposity index by 32.3%, 30.8%, and 41.5%, respectively. A more prominent reduction was observed in the HFD/Dapa + IF-treated rats compared with that in the single-treatment groups. To note, the HFD/IF-treated group showed no significant difference compared to the HFD/Dapa-treated group (*P* > 0.05).

### Effects of Dapa, IF, and their combination on the histopathological alterations, number and surface area of adipocytes induced by HFD in rats

**Fig. 5:**
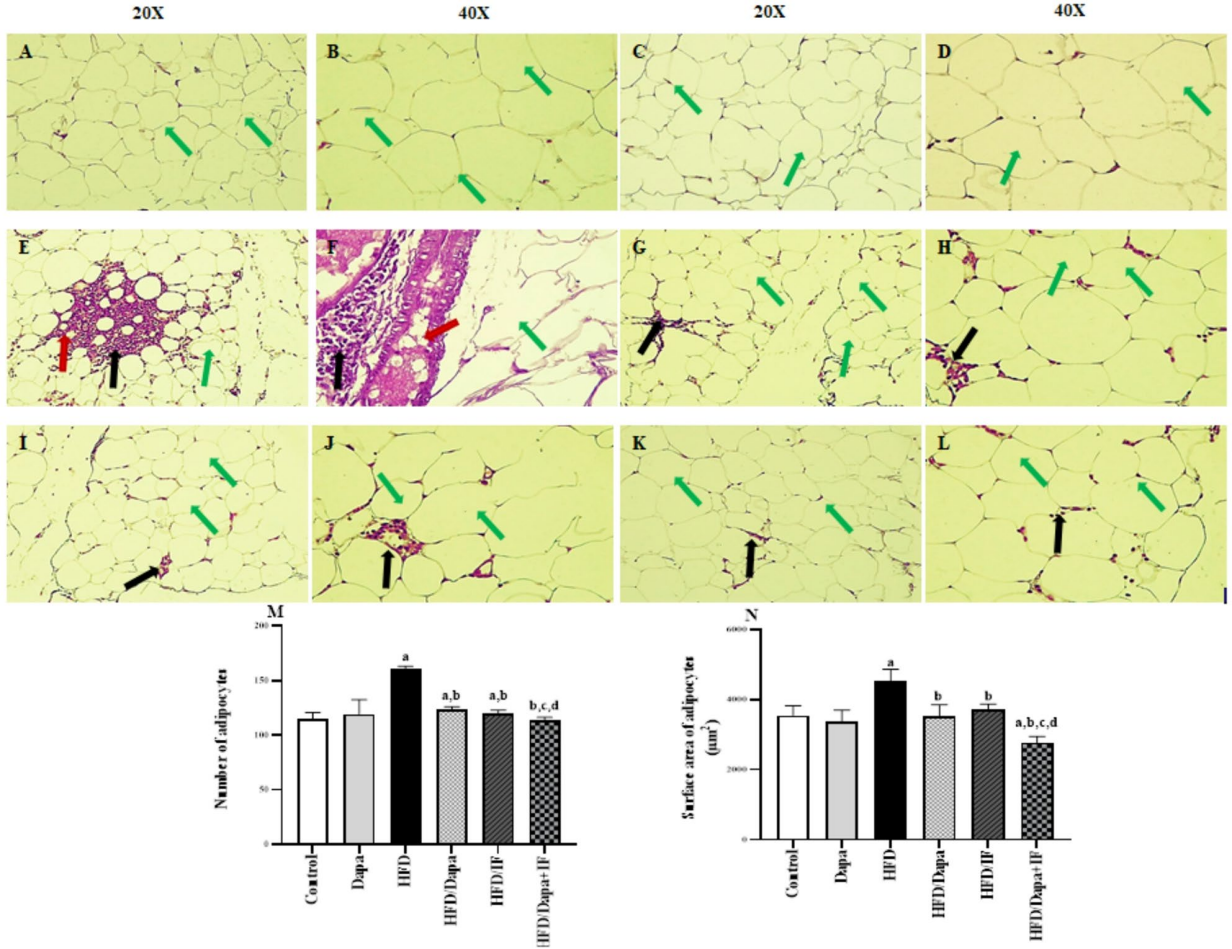
Photomicrographs representing the adipose tissue from the studied groups at week 16 and effects of Dapa, IF, and their combination on the number and surface area of adipocytes in HFD-induced obesity in rats. **A** Photomicrograph of the normal control group (20X) showing normal adipocyte morphology, with uniform, unilocular adipocytes (green arrow) and no signs of inflammation. **B** Photomicrograph of the normal control group (40X) showing a typical adipocytic architecture, with single-vacuole adipocytes (green arrow) and absence of inflammation. **C** and **D** Photomicrographs of the Dapa-control group (20X) and (40X), respectively, showing comparable adipocyte morphology to the normal control group, with no notable changes in adipocyte structure (green arrow) or inflammatory response. **E** Photomicrograph of the HFD group (20X) exhibiting marked adipose tissue pathology, characterized by adipocyte hypertrophy and hypercellularity (green arrow), extensive inflammatory cell infiltration (black arrow), and potential adipocyte necrosis (red arrow). **F** Photomicrograph of the HFD group (40X) exhibiting notable changes, including enlarged adipocytes (green arrow), increased inflammatory cell presence (black arrow), and marked dilated blood vessels (red arrow. **G** and **H** Photomicrographs of the HFD/Dapa group (20X) and (40X), respectively, showing a decreased adipocyte hypertrophy (green arrow) and inflammation (black arrow) in adipose tissue. **I** and **J** Photomicrographs of the HFD/IF group (20X) and (40X), respectively, showing a restoration in normal adipocyte morphology (green arrow), and reduced inflammation. **K** and **L** Photomicrographs of the HFD/Dapa + IF group (20X) and (40X), respectively, showing substantial improvements in adipocyte morphology, with single-vacuole adipocytes along with reduced hypertrophy (green arrow) and decreased inflammation (black arrow). **M** Number of adipocytes [F (5,144) = 197.9, *P* < 0.0001]. **N** Surface area of adipocytes [F (5,144) = 99.90, *P* < 0.0001]. Data are presented as mean ± SD, n = 25. Tukey’s post-hoc test was conducted following ANOVA. ^a^significant from the normal control group, ^b^significant from the HFD group, ^c^significant from the HFD/Dapa, ^d^significant from the HFD/IF. *P* < 0.05 was set as the significance level

The normal control group showed normal adipocyte morphology, with uniform, unilocular adipocytes and no signs of inflammation, suggesting intact adipose tissue structure and function, as shown in Fig. 5A and B. The Dapa-control group exhibited comparable adipocyte morphology to the normal control group, with no notable changes in the adipocyte structure or inflammatory response, indicating a neutral effect of Dapa on adipose tissue structure, as shown in Fig. 5C and D.

Regarding the HFD group, adipocytes exhibited marked adipose tissue pathology, characterized by adipocyte hypertrophy and hypercellularity, extensive inflammatory cell infiltration, potential adipocyte necrosis, and marked dilated blood vessels, indicating a detrimental impact of HFD on adipose tissue health, as shown in Fig. 5E and F. Adipocytes from both the HFD/Dapa (Fig. 5G and H) and HFD/IF-treated groups (Fig. 5I and J) had decreased adipocyte hypertrophy, with no signs of fatty deposits, and reduced inflammation compared to the HFD group.

Concerning the HFD/Dapa + IF group (Fig. 5K and L), adipocytes showed substantial improvements in morphology, with a remarkable decrease in size, organized and well-arranged structures with distinct cell membranes, reduced hypertrophy, and decreased inflammation, suggesting a potential synergistic effect of the combined therapy in mitigating HFD-induced adipose tissue damage.

As depicted in Fig. 5M and N, the number and surface area of adipocytes were significantly increased in the HFD group compared to the normal control group by 40.7% and 27.5%, respectively. Treatment with Dapa, IF, or their combination mitigated the effect of HFD, as evidenced by decreasing the number of adipocytes by 23.6%, 25.6% and 29.3%, respectively, and reducing the surface area by 22.4%,17.8%, and 39.3%, respectively.

Interestingly, the HFD/Dapa + IF-treated rats normalized the number of adipocytes, while showing a more pronounced reduction in the surface area of adipocytes compared to the normal control group. Of note, HFD/IF-treated group showed no significant difference in the number or surface area of adipocytes compared to the HFD/Dapa-treated group (*P* > 0.05).

### Effects of Dapa, IF, and their combination on liver histology, liver index, ALT and AST activities in HFD-induced obesity in rats

The impact of HFD and different interventions on the liver was assessed using liver histopathology, liver index (liver weight/final body weight) as a marker of enlarged liver, and serum ALT and AST activities as sensitive indicators of hepatocellular damage, secondary to fat accumulation.

**Fig. 6:**
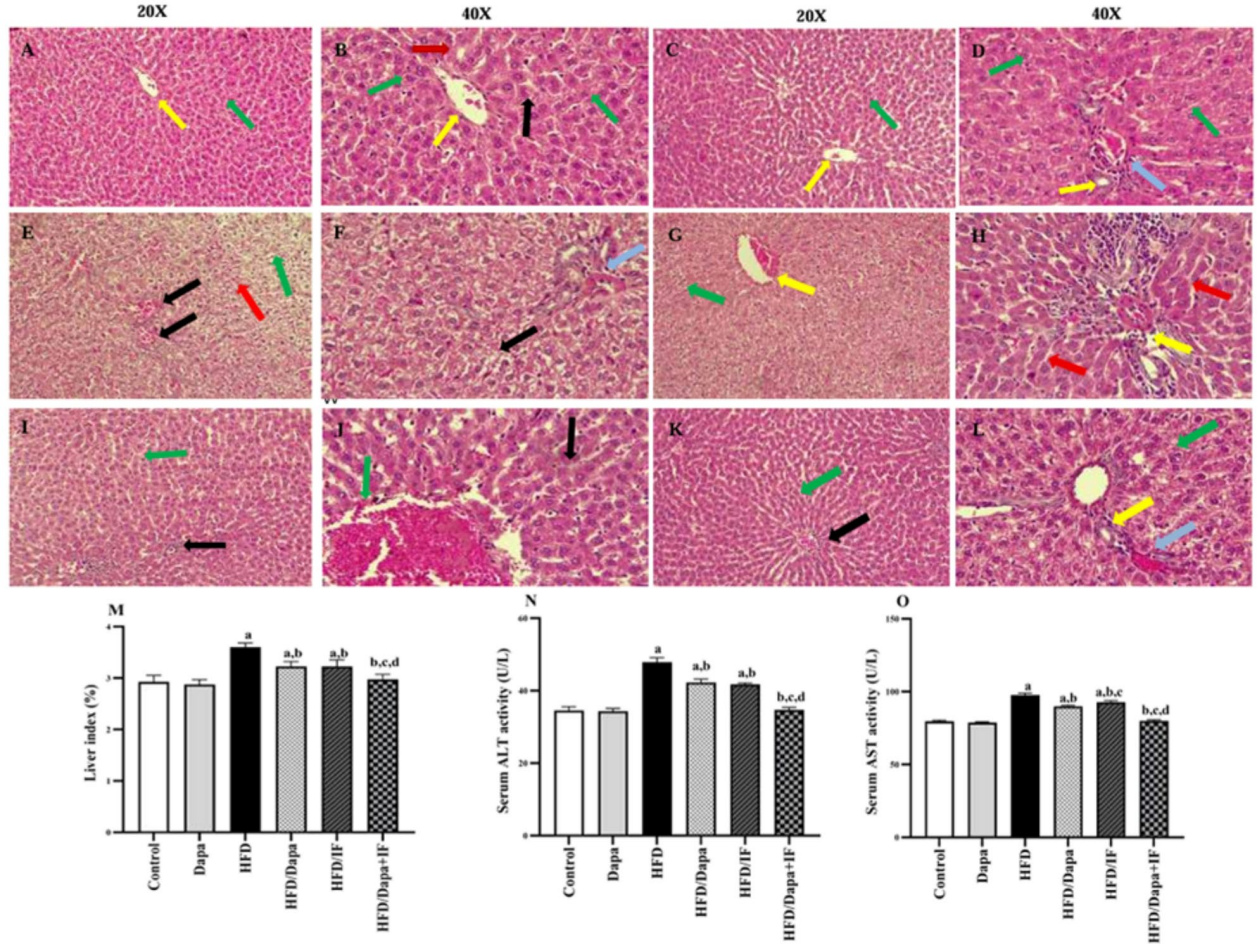
Photomicrographs representing the liver tissue from the studied groups at week 16 and effects of Dapa, IF, and their combination on liver index and serum liver enzymes in HFD-induced obesity in rats. **A** Photomicrograph of the normal control group (20X) showing normal classic hepatic lobule (green arrow) with normal central vein (yellow arrow). **B** Photomicrograph of the normal control group (40X) displaying normal hepatocyte morphology, characterized by vesicular nuclei with prominent nucleoli and acidophilic cytoplasm (green arrow), with some binucleated cells (black arrow). The central vein (yellow arrow) and blood sinusoids lined with flat endothelial cells (red arrow) are normal, separating the radiating hepatocyte plates. **C** Photomicrograph of the Dapa-control group (20X) showing normal hepatic morphology, comparable to the normal control group, with a typical lobule (green arrow) and central vein (yellow arrow) **D** Photomicrograph of the Dapa-control group (40X) showing normal hepatocytes with vesicular nuclei and acidophilic cytoplasm (green arrow) arranged around portal tracts containing a hepatic artery, portal vein (blue arrow), and normal bile ducts lined with cuboidal epithelium (yellow arrow). **E** Photomicrograph of the HFD group (20X) exhibiting significant histological changes, including hepatocyte vacuolation with many fat droplets (green arrow), congested blood vessels (black arrow), and dilated congested sinusoids (red arrow). **F** Photomicrograph of the HFD group (40X) showing vacuolated hepatocytes with accumulation of fat droplets (green arrow), thickened dilated blood vessels of portal area (black arrow), hepatocellular ballooning, and apoptotic nuclei with pyknosis (blue arrow). **G** Photomicrograph of the HFD/Dapa group (20X) revealing some restoration of normal hepatic architecture, with an apparent classic hepatic lobule near normal (green arrow) and a mildly dilated congested central vein in the middle (yellow arrow). **H** Photomicrograph of the HFD/Dapa group (40X) showing the portal tract appearing normal with mildly thickened blood vessels (yellow arrow), and most hepatocytes normally arranged in radiating plates separated by blood sinusoids (red arrow). **I** Photomicrograph of the HFD/IF group (20X) showing restoration of normal hepatic lobule architecture, with hepatocytes arranged in radiating plates separated by blood sinusoids (green arrow) and preserved portal tract structure (black arrow). **J** Photomicrograph of the HFD/IF group (40X) exhibiting a normal-appearing classic lobule, a mildly dilated central vein (green arrow), and mild residual steatosis in some hepatocytes (red arrow). Hepatocytes display typical features, including vesicular nuclei and acidophilic cytoplasm (black arrow). **K** Photomicrograph of the HFD/Dapa + IF group (20X) showing marked restoration of hepatic lobule architecture, with hepatocytes in radiating plates separated by blood sinusoids (green arrow) and a normal central vein (black arrow). **L** Photomicrograph of the HFD/Dapa + IF group (40X) showing significant restoration of normal hepatic architecture, with normal portal tracts containing a hepatic artery and portal vein (blue arrow), normal bile ducts lined with cuboidal epithelium (yellow arrow), and hepatocytes arranged in radiating plates separated by blood sinusoids (green arrow). **M** Liver index (liver weight / final body weight × 100), F (5,18) = 26.95, *P* < 0.0001, n = 4. N) Serum alanine aminotransferase (ALT) activity [F (5,30) = 256.1, *P* < 0.0001], n = 6. **O** Serum aspartate aminotransferase (AST) activity [F (5,30) = 557.9, *P* < 0.0001], n = 6. Data are presented as mean ± SD. Tukey’s post-hoc test was conducted following ANOVA. ^a^significant from the normal control group, ^b^significant from the HFD group, ^c^significant from the HFD/Dapa, ^d^significant from the HFD/IF. *P* < 0.05 indicated statistical significance

In the histology examination, the normal control group showed normal classic hepatic lobule with normal central vein, normal hepatocyte morphology, characterized by vesicular nuclei with prominent nucleoli and acidophilic cytoplasm, with some binucleated cells. The central vein and blood sinusoids lined with flat endothelial cells are normal, separating the radiating hepatocyte plates, as shown in Fig. 6A and B. The Dapa-control group (Fig. 6C and D) exhibited comparable hepatic morphology with no notable changes from the normal control group.

Regarding the HFD group (Fig. 6E and F), liver tissue showed significant histological changes, including hepatocyte vacuolation with many fat droplets (steatosis), congested blood vessels, dilated congested sinusoids, hepatocellular ballooning, and apoptotic nuclei with pyknosis. As depicted in Fig. 6G and H, hepatocytes from the HFD/Dapa-treated group revealed some restoration of normal hepatic architecture with a mildly dilated congested central vein in the middle, and most hepatocytes normally arranged in radiating plates separated by blood sinusoids, indicating a return to normal liver histology. The HFD/IF-treated group showed partial restoration of normal hepatic lobule architecture, a mildly dilated central vein, and mild residual steatosis in some hepatocytes, as shown in Fig. 6I and J.

Concerning the HFD/Dapa + IF-treated group (Fig. 6K and L), hepatocytes showed marked improvements and restoration in morphology with normal portal tracts containing a hepatic artery and portal vein, normal bile ducts lined with cuboidal epithelium, and hepatocytes arranged in radiating plates separated by blood sinusoids.

The liver index was significantly increased in the HFD group by 24.1% relative to the normal control group (Fig. 6M), indicating hepatomegaly. Dapa, IF, and their combination reduced the liver index by 10.4%, 10.4%, and 17.4%, respectively, compared to the HFD group. The reduction was similar in the HFD/IF-treated and the HFD/Dapa-treated groups (*P* > 0.05). A more prominent reduction in the liver index was observed in the HFD/Dapa + IF-treated rats compared with that in the single-treatment groups, reaching normalization.

Serum ALT and AST activities were significantly elevated in the HFD group by 38.8% and 22.6%, respectively, compared to the normal control group (Figs. 6N and O), indicating liver cell damage secondary to steatosis. Administration of Dapa, IF, or their combination attenuated serum ALT activity by 11.7%, 12.8%, and 27.5%, respectively, and serum AST activity by 8%, 5%, and 18.1%, respectively, compared with their activities in the HFD group. Treatment with Dapa + IF combination normalized both enzyme activities, with a more noticeable reduction was observed compared to the other intervention groups. The HFD/Dapa-treated group exhibited significantly lower AST activity compared to the HFD/IF-treated group, while there was no significant difference in serum ALT activity between the two groups.

### Effects of Dapa, IF, and their combination on fasting serum glucose and lipid profile in HFD-induced obesity in rats

**Fig. 7:**
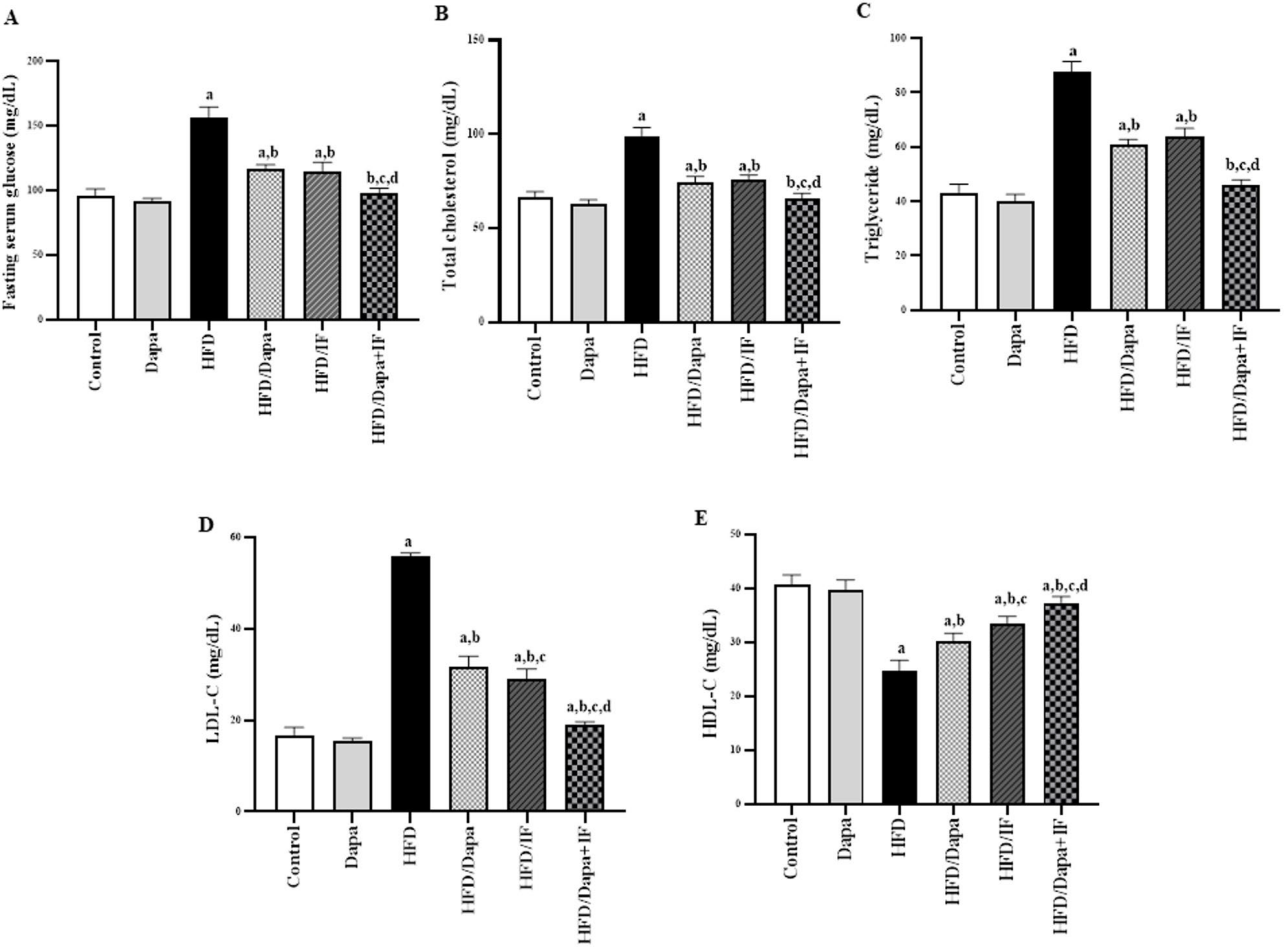
Effects of Dapa, IF, and their combination on the fasting serum glucose and lipid profile in HFD-induced obesity in rats. Data are displayed as mean ± SD, normal control group: n = 8; other groups: n = 10. Following ANOVA, Tukey’s post-hoc test was conducted. **A** Serum glucose [F (5,52) = 208.8, *P* < 0.0001]. **B** Serum total cholesterol [F (5,52) = 183.1, *P* < 0.0001]. **C** Serum triglycerides [F (5,52) = 394.4, *P* < 0.0001]. **D** Serum low-density lipoprotein-cholesterol (LDL-C) [F (5,52) = 934.7, *P* < 0.0001]. **E** Serum high-density lipoprotein-cholesterol (HDL-C) [F (5,52) = 131.3, *P* < 0.0001]. ^a^significant from the normal control group, ^b^significant from the HFD group, ^c^significant from the HFD/Dapa, ^d^significant from the HFD/IF. The significance level was set at *P* < 0.05

Compared to the normal control group, the HFD group exhibited a significant increase in fasting serum glucose levels by 66.7% (Fig. 7A). Serum glucose levels were significantly lower in the HFD/Dapa, HFD/IF, and HFD/Dapa + IF fasted rats than those in the HFD group by 27.5%, 28.1%, and 38.8%, respectively. Notably, the HFD/Dapa + IF-treated group exhibited a more pronounced reduction in glucose levels, reaching normalization, compared to the HFD/Dapa and HFD/IF-treated groups. However, there was no significant difference in the fasting serum glucose levels between the latter two groups *(P* > *0.05).*

Regarding the lipid profile, serum total cholesterol, triglycerides, and LDL-C levels were significantly increased in the HFD group by 50%, 104.7%, and 232.1%, respectively, whereas serum HDL-C was significantly reduced by 36.9% compared to the normal control group (Fig. 7B–E). Dapa, IF, or their combination reduced total cholesterol by 25.3%, 24.2%, and 34.3%; triglycerides by 31.8%, 28.4%, and 48.3%; and LDL-C levels by 43%, 48.2%, and 66.1%, respectively, compared with levels in the HFD group. Meanwhile, HDL-C levels were significantly increased in the HFD/Dapa, HFD/IF, and HFD/Dapa + IF-treated groups by 17.9%, 30.8%, and 44.5%, respectively, compared to the HFD group.

The HFD/IF-treated group had significantly lower LDL-C and higher HDL-C levels than the HFD/Dapa-treated group. However, there were no significant differences in the total cholesterol and triglyceride levels between the two groups. Noteworthy, the combination of Dapa and IF exerted a more prominent impact on improving the lipid profile than a single treatment did, reaching normalization of total cholesterol and triglyceride levels.

### Effects of Dapa, IF, and their combination on serum insulin, leptin, adiponectin, POMC, and PYY levels in HFD-induced obesity in rats

**Fig. 8:**
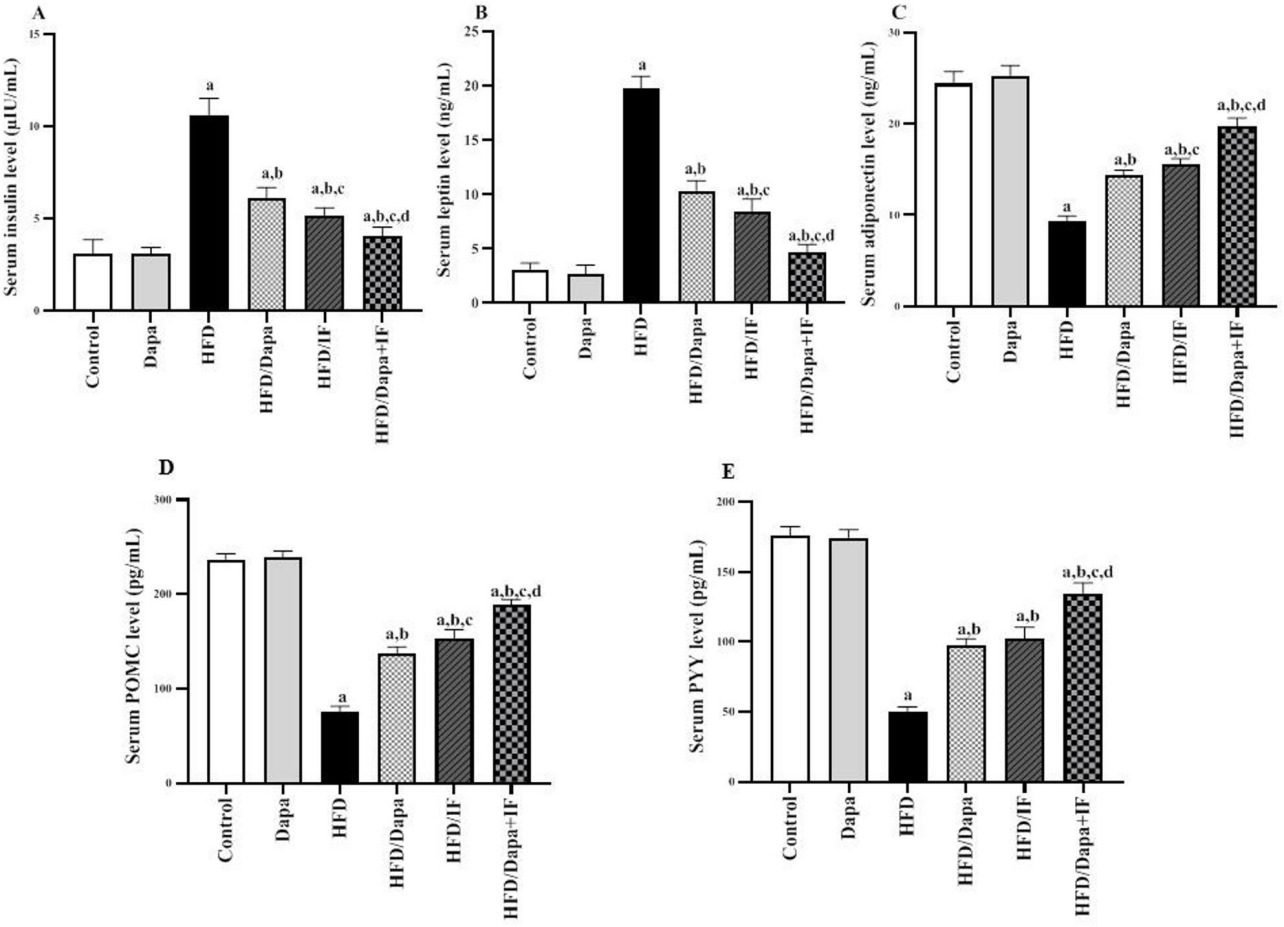
Effects of Dapa, IF, and their combination on serum insulin, leptin, adiponectin, and appetite hormones in HFD-induced obesity in rats. **A** Serum insulin [F (5,52) = 202.2, *P* < 0.0001]. **B** Serum leptin [F (5,52) = 470.8, *P* < 0.0001]. **C** Serum adiponectin [F (5,52) = 441.8, *P* < 0.0001]. **D** Serum proopiomelanocortin (POMC) [F (5,52) = 719.0, *P* < 0.0001]. E) Serum peptide YY (PYY) [F (5,52) = 595.9, *P* < 0.0001]. Data are displayed as mean ± SD, normal control group: n = 8; other groups: n = 10. Following ANOVA, Tukey’s post-hoc test was conducted. ^a^significant from the normal control group, ^b^significant from the HFD group, ^c^significant from the HFD/Dapa group, ^d^significant from the HFD/IF group. The significance level was set at *P* < 0.05

Serum insulin and leptin levels were increased in the HFD-induced obese rats by 3.2- and 6.4-fold, respectively, compared with levels in the normal control group, as depicted in Fig. 8A and B. The HFD/Dapa, HFD/IF, and HFD/Dapa + IF-treated groups showed a marked decrease in insulin by 42.5%, 51.9%, and 61.3%, respectively, and in leptin levels by 47.7%, 56.9%, and 76.1%, respectively, compared with those in the HFD group. In contrast, serum adiponectin levels (Fig. 8C) were significantly decreased in the HFD group by 61.9% in comparison with the normal control group. The HFD/Dapa, HFD/IF, and HFD/Dapa + IF-treated groups exhibited a significant increase in adiponectin levels by 53.8%, 66.7%, and 111.8%, respectively, compared to the HFD group.

While the HFD/IF-treated group showed significantly lower insulin and leptin levels and higher adiponectin levels in comparison with the HFD/Dapa-treated group, the impact was more pronounced in the HFD/Dapa + IF-treated group compared to the other treated groups.

Regarding appetite hormones (Fig. 8D and E), serum POMC and PYY levels were noticeably decreased in the HFD-induced obese rats by 68.3% and 71.7%, respectively, when compared with the normal control group. Rats treated with Dapa, IF, or their combination showed a significant increase in POMC by 82.9%, 104%, and 152.1%, respectively, and in PYY levels by 96.6%, 105.6%, and 170.7%, respectively, in comparison with the HFD group.

Notably, serum POMC levels were significantly higher in the HFD/IF-treated rats than levels in the HFD/Dapa-treated group, while PYY levels were not significantly different between the two groups (*P* > 0.05). Interestingly, the levels of both markers were significantly elevated in the HFD/Dapa + IF-treated group compared with those in the other treated groups.

### Effects of Dapa, IF, and their combination on adipose tissue p-AMPK, SIRT1, and SIRT7 expression in HFD-induced obesity in rats

**Fig. 9:**
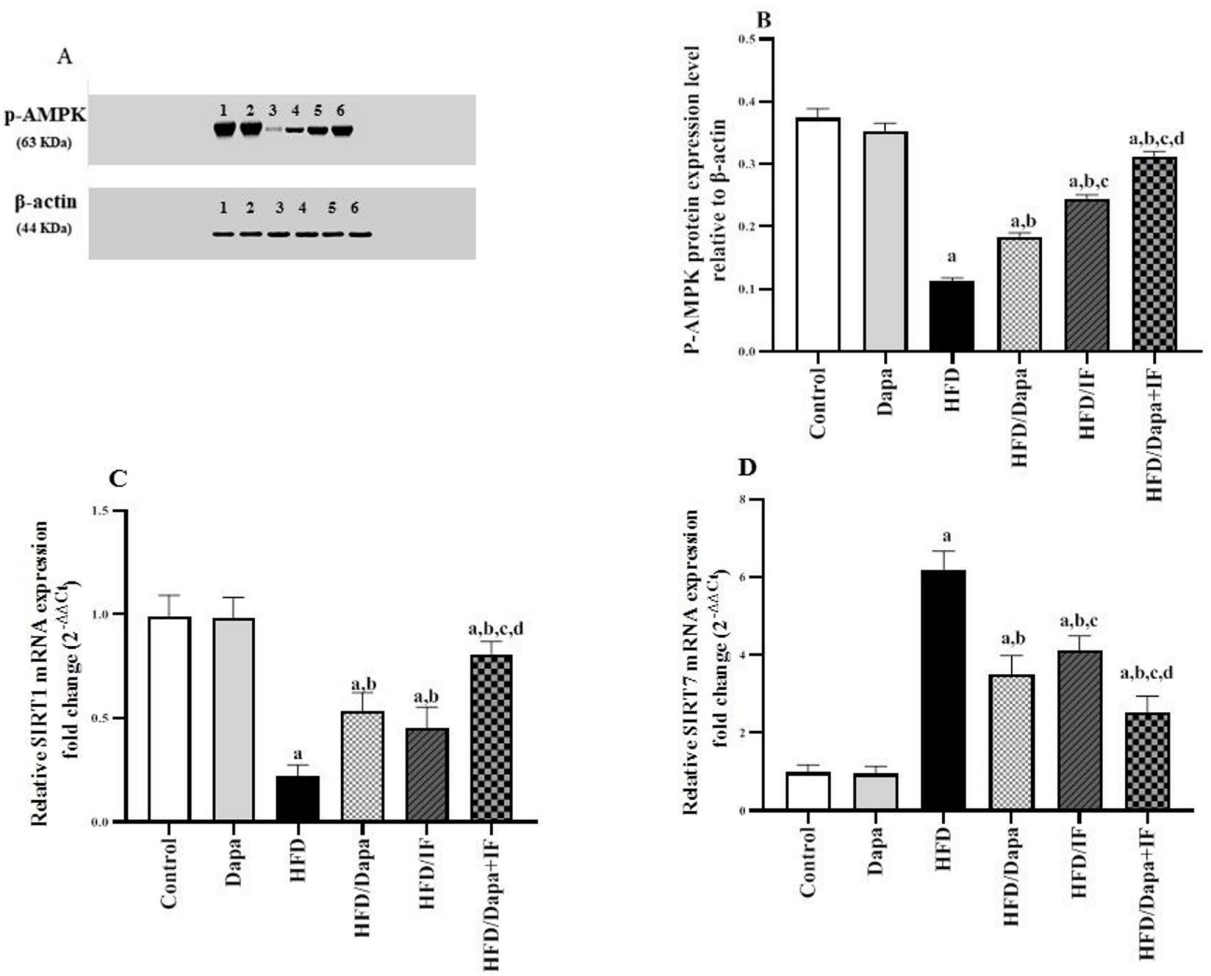
Effects of Dapa, IF, and their combination on adipose tissue p-AMPK, SIRT1, and SIRT7 expression in HFD-induced obesity in rats. **A** Cropped western blot images of phosphorylated-AMP-activated protein kinase (p-AMPK) and the housekeeping gene β-actin in different studied groups: 1: control, 2: Dapa, 3: HFD, 4: HFD/Dapa, 5: HFD/IF, and 6: HFD/Dapa + IF. **B** The mean ± SD of p-AMPK expression relative to β-actin [F (5,12) = 331.0, *P* < 0.0001], n = 3, **C** Relative sirtuin 1 (SIRT1) mRNA expression [F (5,52) = 77.06, *P* < 0.0001], **D** Relative sirtuin 7 (SIRT7) mRNA expression [F (5,52) = 113.2, *P* < 0.0001]. Data of SIRT1 and SIRT7 are displayed as mean ± SD, normal control group: n = 8; other groups: n = 10. Fold change was calculated using the 2^−∆∆Ct^ formula. Following ANOVA, Tukey’s post-hoc test was conducted. ^a^significant from the normal control group, ^b^significant from the HFD group, ^c^significant from the HFD/Dapa group, ^d^significant from the HFD/IF group. The significance level was set at *P* < 0.05

Figure 9 displays the p-AMPK protein levels, along with the mRNA expression levels of SIRT1 and SIRT7 in the adipose tissue of different studied groups.

The expression of p-AMPK protein in the adipose tissue of HFD-induced obese rats was significantly decreased by 70.3% compared to the normal control group, as shown in Fig. 9A and B. Compared to the HFD group, the HFD/Dapa, HFD/IF, and HFD/Dapa + IF-treated rats exhibited a significant increase in p-AMPK expression by 63.6%, 118.2%, and 181.8%, respectively.

Notably, the HFD/IF-treated group exhibited a significantly higher p-AMPK protein expression than that in the HFD/Dapa-treated group. Compared with the rats treated with Dapa or IF alone, the HFD/Dapa + IF-treated rats presented with a more prominent elevation of p-AMPK protein.

Compared to the normal control group, SIRT1 expression was significantly decreased by 77.8%, whereas SIRT7 expression was significantly increased by 526.3% in the HFD-induced obese rats, as shown in Fig. 9C and D, respectively. Notably, the HFD/Dapa, HFD/IF, and HFD/Dapa + IF-treated rats exhibited a marked increase in SIRT1 expression levels by 140.9%, 104.5%, and 263.6%, respectively, and a significant decrease in SIRT7 expression levels by 43.5%, 30.65%, and 59.7%, respectively, when compared with the HFD group.

While the HFD/IF-treated group showed no significant difference in SIRT1 expression levels (*P* > 0.05), it exhibited noticeably lower SIRT7 expression levels than those in the HFD/Dapa-treated group. To note, rats receiving combined Dapa/IF treatment exhibited significantly higher SIRT1 and lower SIRT7 expression levels than those receiving either treatment alone.

### Effects of Dapa, IF, and their combination on adipose tissue expression of clock genes in HFD-induced obesity in rats

**Fig. 10:**
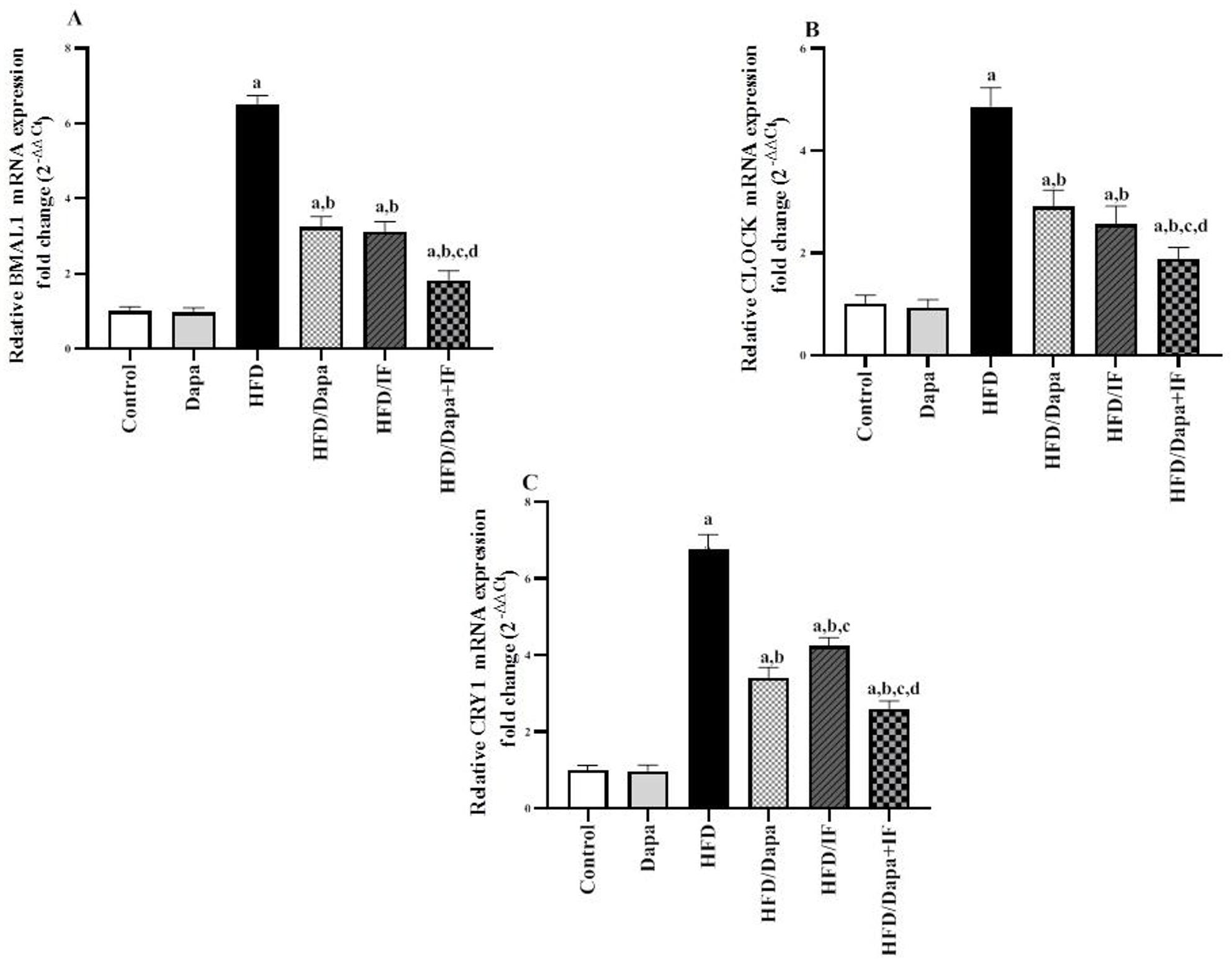
Effects of Dapa, IF, and their combination on adipose tissue BMAL1, CLOCK, and CRY1 expression in HFD-induced obesity in rats. **A** Relative brain and muscle ARNT-like protein 1 (BMAL1) mRNA expression [F (5,52) = 847.7, *P* < 0.0001]. **B** Relative circadian locomotor output cycles kaput (CLOCK) mRNA expression [F (5,52) = 285.8, *P* < 0.0001]. **C** Relative cryptochrome 1 (CRY1) mRNA expression [F (5,52) = 890.0, *P* < 0.0001]. Data are displayed as mean ± SD, normal control group: n = 8; other groups: n = 10. Fold change was calculated using the 2^−∆∆Ct^ formula. Following ANOVA, Tukey’s post-hoc test was conducted. ^a^significant from the normal control group, ^b^significant from the HFD group, ^c^significant from the HFD/Dapa group, ^d^significant from the HFD/IF group. The significance level was set at *P* < 0.05

The mRNA expression levels of clock genes BMAL1, CLOCK, and CRY1 are presented in Fig. 10. Administration of HFD significantly increased the gene expression of BMAL1, CLOCK, and CRY1 by 6.5, 4.9, and 6.8 folds, respectively, compared to the normal control group. Notably, HFD-induced obese rats receiving Dapa, IF, or their combination exhibited a significant decrease in BMAL1 by 50.8%, 52.3%, and 72.3%; CLOCK by 40.8%, 46.9%, and 61.2%; and CRY1 expression levels by 50%, 38.2%, and 61.8%, respectively, when compared with the HFD alone group.

The HFD/Dapa-treated group had much lower levels of CRY1 expression than those in the HFD/IF-treated group. However, there were no significant differences in the levels of BMAL1 and CLOCK expression between the two groups (*P* > 0.05). In the HFD/Dapa + IF combination group, the reduction of BMAL1, CLOCK, and CRY1 expression levels was more pronounced than in the single-treatment groups.

#### Effects of Dapa, IF, and their combination on gut microbiome composition and relative abundance, serum SCFAs, and adipose GPR43 receptor expression in HFD-induced obesity in rats

**Fig. 11:**
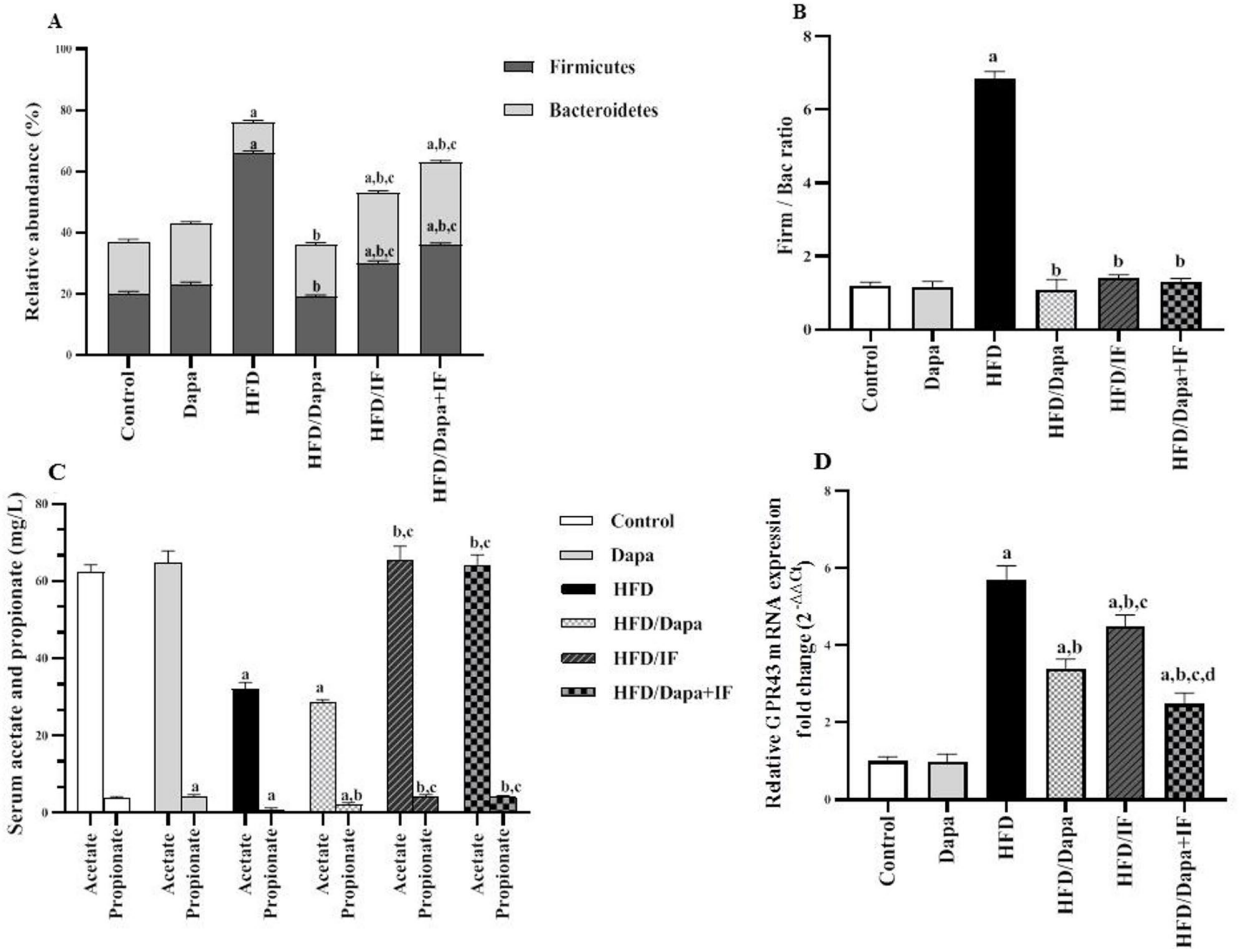
Effects of Dapa, IF, and their combination on the relative abundance of the gut microbiome and SCFA/GPR43 receptor signaling in HFD-induced obesity in rats. **A** Representative stacked column for the relative abundance of gut microbiota at the phylum level [Firmicutes F (5,12) = 126.3, *P* < 0.0001 and Bacteroidetes F (5,12) = 43.71, *P* < 0.0001] in the studied groups, n = 3. **B** Firm /Bac ratio [F (5,12) = 1449, *P* < 0.0001] in the studied groups displayed as mean ± SD, n = 3. **C** Serum levels of the SCFAs (acetate and propionate) displayed as mean ± SD, n = 6. For acetate, F (5,30) = 324.6, *P* < 0.0001, for propionate, F (5,30) = 178.1, *P* < 0.0001. **D** Relative G-protein-coupled receptor 43 (GPR43) mRNA expression in the adipose tissue [F (5,52) = 483.1, *P* < 0.0001] presented as mean ± SD, normal control group: n = 8; other groups: n = 10. Tukey’s post-hoc test was conducted following ANOVA. ^a^significant from the normal control group, ^b^significant from the HFD group, ^c^significant from the HFD/Dapa group, ^d^significant from the HFD/IF group. The significance level was set at *P* < 0.05

To evaluate the major composition and the relative abundance of gut microbiota in rats’ intestinal content at the class, order, and genus levels, we focused on two main phyla: Firmicutes and Bacteroidetes (Fig. 11A–B). The Krona charts showing the relative abundance of the gut microbiome at the class, order, genus levels in rats’ intestinal content in different studied groups are given in Supplementary Figures S1, S2, and S3.

As depicted in Fig. 11A, the Dapa-control group showed no marked difference regarding the relative abundance of the Firmicutes and Bacteroidetes compared to the normal control group (*P* > 0.05). Relative to the normal control rats, the HFD group had a significant increase in the relative abundance of the Firmicutes and a reduction in the Bacteroidetes levels. Treatment of the HFD group with Dapa, IF, or both resulted in a significant increase in the phylum Bacteroidetes and a decrease in Firmicutes.

Notably, the HFD/IF and the HFD/Dapa + IF-treated groups showed a noticeably higher relative abundance of both Firmicutes and Bacteroidetes compared to the HFD/Dapa-treated group. However, there was no significance difference between the HFD/IF and HFD/Dapa + IF-treated groups regarding the abundance of these two phyla (*P* > 0.05).

The Firmicutes to Bacteroidetes ratio (Firm/Bac ratio) is considered a strong marker of obesity. This ratio was significantly increased in the HFD-induced obese rats compared to the normal controls. The HFD/Dapa, HFD/IF, and HFD/Dapa + IF-treated groups showed a significant decrease in the Firm/Bac ratio compared to the HFD group. Interestingly, the three treatment groups normalized this ratio, as shown in Fig. 11B.

We then evaluated the serum levels of SCFAs: acetate, propionate, and butyrate in the studied groups. Notably, serum butyrate levels were undetectable in all studied groups. The mass spectra of serum acetate and propionate in different groups are given in Supplementary Figures S4 and S5. Additionally, samples were re-analyzed using targeted GC–MS SIM to enhance analytical sensitivity. Representative chromatograms confirmed the presence of acetate and propionate in serum samples of different studied groups (Supplementary Figures S6 and S7), whereas butyrate was specifically monitored but remained undetectable in all groups, with signals below the previously established LOD (0.14 mg/L). These findings indicate very low circulating levels of butyrate rather than limitations related to assay sensitivity or sample handling.

Compared to the normal control group, the Dapa-control group showed no significant differences regarding serum acetate and propionate levels (*P* > 0.05). The HFD group showed a marked decrease in serum acetate and propionate levels by 48.6% and 78.4%, respectively, compared with levels in the normal control group (Fig. 11C). In the HFD/Dapa-treated group, serum propionate was increased by 162.5% compared to the HFD group, whereas serum acetate was slightly decreased (by 11.3%); however, it did not reach the statistical significance (*P* > 0.05). Comparing the HFD/IF and HFD/Dapa + IF-treated groups with the HFD group revealed a significant increase in serum acetate by 104.7% and 103.4%, respectively, and in propionate levels by 412.5% and 400%, respectively, reaching normalization. These levels were significantly higher than those in the HFD/Dapa-treated group.

Regarding the GPR43 receptor (Fig. 11D), its mRNA expression in adipose tissue was markedly increased by 5.7-fold in the HFD group compared to the normal control group. The HFD/Dapa, HFD/IF, and HFD/Dapa + IF-treated groups showed a significant decrease in GPR43 expression levels by 40.4%, 21.1%, and 56.1%, respectively, compared with levels in the HFD group.

Compared to the HFD/IF-treated group, the HFD/Dapa-treated group demonstrated much lower levels of GPR43 expression. Interestingly, rats receiving combined Dapa/IF treatment showed a more profound reduction in GPR43 expression compared with rats experiencing Dapa treatment or IF alone.

### Correlation between gut microbiome and adipose tissue parameters

**Fig. 12:**
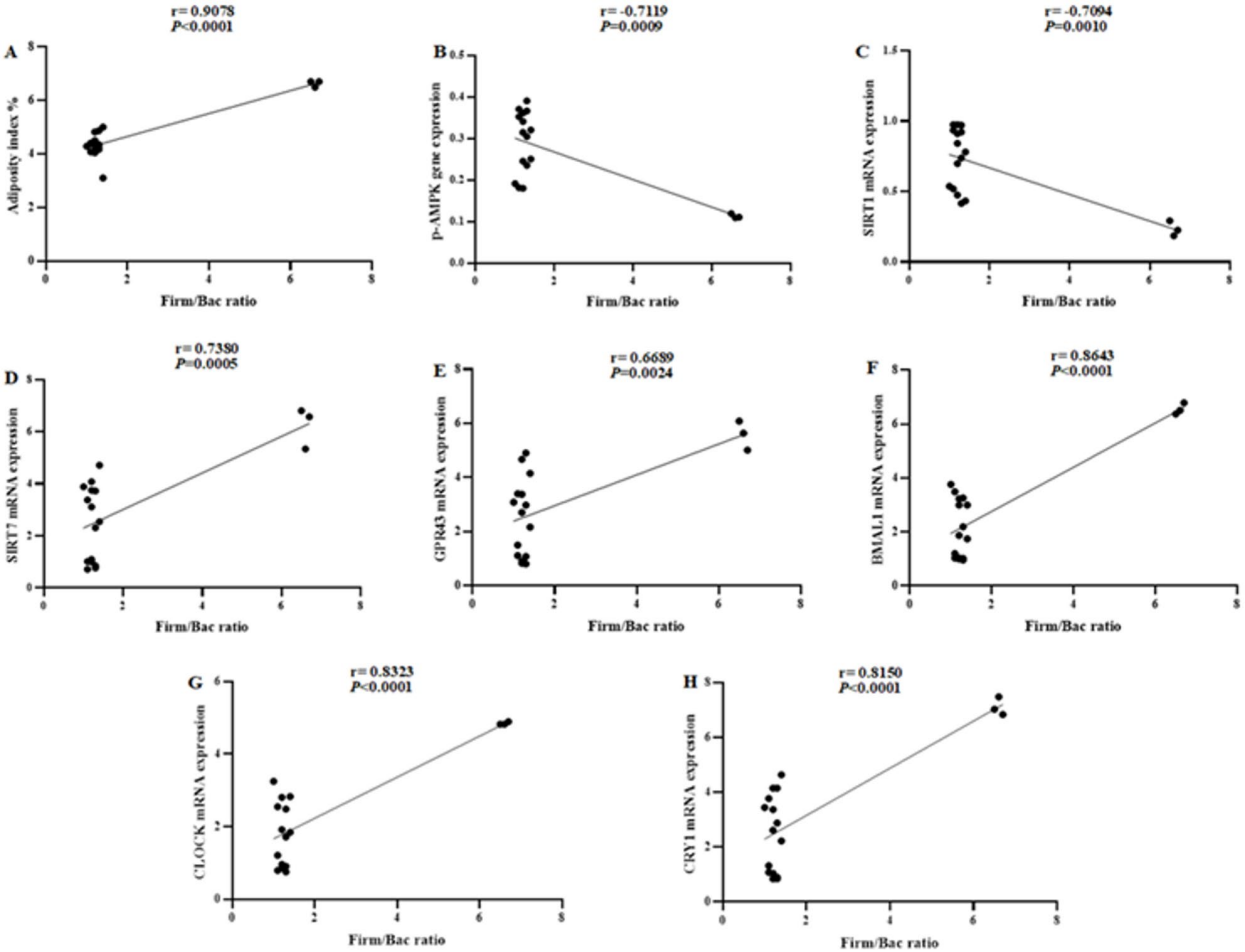
Correlation between gut microbiome and adipose tissue parameters. Correlation was done in all the studied groups collectively (total n = 18). The groups’ data were aligned vertically and the parameters horizontally. Each raw contained the parameters' values from the same rat

We investigated the correlation between the Firm/Bac ratio and the adiposity index, along with parameters measured in adipose tissue, including p-AMPK, SIRT1, SIRT7, GPR43, BMAL1, CLOCK, and CRY1 (Fig. 12A–H). The Firm/Bac ratio was positively correlated with the adiposity index and adipose tissue levels of SIRT7, GPR43, and clock genes, while inversely correlated with p-AMPK and SIRT1. The correlations between Firmicutes or Bacteroidetes individual data and adipose tissue parameters are presented in Supplementary Figures S8 and S9.

## Discussion

Innovative and affordable treatment options are required to combat the obesity epidemic and its detrimental impact on healthcare expenditures, productivity, and economic development. To our knowledge, this study is the first to test the repercussion of combining Dapa and IF in treating experimental obesity. Furthermore, it is also the first to report the impact of Dapa on key circadian and metabolic markers such as GPR43, BMAL1, CLOCK, CRY1, and SIRT7, as well as its selective effect on serum propionate levels. The current literature indicates that Dapa offers multiple metabolic advantages beyond glucose reduction like cardioprotective effects and improved insulin sensitivity [38], though its mechanisms for weight reduction are not well delineated. IF has been shown to reset metabolic profiles and improve obesity-related mitochondrial dysfunction, positioning it as a potentially safe weight reduction method [39]. Thereby, this study explored the impact of Dapa, IF, and their combination on weight reduction and metabolic derangement in rats with HFD-induced obesity, focusing on sirtuins, clock genes, appetite-regulating peptides, gut microbiome, and SCFAs.

The HFD-induced obesity model in rats closely mimics the pathophysiological characteristics of human obesity, including insulin resistance, dyslipidemia, and low-grade inflammation, providing insights into the complex molecular underpinnings [40]. In this study, HFD-fed rats had higher body weight, BMI, and other anthropometric measures, along with abnormal lipid profile, increased serum insulin and leptin, reduced adiponectin levels, and a higher adiposity index. These findings support earlier research [41–44] and are consistent with features of human obesity.

Obesity usually arises from a long-term imbalance between energy intake and output. Nutrient abundance is converted through metabolic pathways into ATP, lowering the AMP/ATP ratio and suppressing the energy sensor, AMPK, in adipocytes. In this study, the HFD group had lower p-AMPK in adipose tissue, consistent with previous results [45], indicating increased energy storage. Indeed, body weight gain has been shown to correlate with reduced AMPK levels [8]. Higher BMI is associated with diminished AMPK signaling and reduced expression of uncoupling protein 1 (UCP1), leading to reduced BAT activity and decreased thermogenesis [8].

p-AMPK regulates energy metabolism and inhibits adipogenesis through multiple mechanisms, including SIRT1 activation in nutrient deficiency states [10]. In our study, abnormal adipogenesis was confirmed by an elevated adiposity index in the HFD group. This picture could be attributed to reduced p-AMPK/SIRT1 expression in the rat adipose tissue, as demonstrated by our findings. These results mirror previous reports demonstrating the downregulation of the p-AMPK/SIRT1/peroxisome proliferator-activated receptor gamma coactivator 1-alpha (PGC-1α) trajectory in the HFD-induced obesity rat model [46]. Indeed, reduced SIRT1 expression increases adipogenesis and decreases free fatty acid mobilization in the visceral WAT, favoring energy storage [13]. Mechanistically, the suppression of the p-AMPK/SIRT1 signaling pathway elevates the levels of key proteins entangled in lipogenesis, including fatty acid synthase (FAS) and sterol regulatory element-binding protein-1c (SREBP-1c), promoting fatty acid synthesis and triglyceride accumulation [47]. Additionally, SIRT1 deficiency leads to hyperacetylation of peroxisome proliferator-activated receptor gamma (PPARγ) in SIRT1 adipocyte-specific knockout mice, resulting in diminished browning in WAT, hence inducing adipogenesis [48].

In addition, glucose intolerance, as indicated in the HFD group by hyperglycemia, increased insulin, and reduced adiponectin levels, could be partly attributed to SIRT1 depletion, which impairs insulin-stimulated glucose uptake and alters GLUT4 translocation in adipocytes, contributing to the development of insulin resistance [49, 50]. Decreased SIRT1 signaling contributes to reduced adiponectin levels in obesity [51]. On the other hand, elevated SIRT1 exerts beneficial effects on insulin and glucose intolerance [13, 50].

In contrast to SIRT1, SIRT7 was upregulated in adipose tissue from the HFD group. This result aligns with prior research showing that SIRT7 is markedly elevated in the adipose tissue of obese individuals [52] and is associated with adipogenesis activation [14]. Indeed, obesity is linked to SIRT1 downregulation and SIRT7 upregulation, favoring increased adipogenesis [11]. This could be explained based on that SIRT7 directly inhibits the autocatalytic activation of SIRT1, thereby promoting adipogenesis [52, 53].

Herein, the imbalance of SIRT1 and SIRT7 favored increased adipogenesis. Aberrant adipogenesis impairs the secretion of adipokines, which are the critical mediators between the risk factors of metabolic syndrome and obesity [54]. The elevated leptin levels observed in the HFD group could be attributed to excessive adiposity, which leads to long-term leptin elevation. This process desensitizes leptin receptors in the brain and alters leptin transport across the blood–brain barrier, indicating the potential onset of leptin resistance [55].

In addition, excessive adiposity conferred by HFD administration for 16 weeks led to an aberrant lipid profile. Gaining weight is linked to increased cholesterol production and, consequently, higher VLDL and LDL-C levels in the bloodstream via SREBP-2 overexpression [56]. Intriguingly, SIRT7 overexpression upregulates cluster of differentiation 36 in WAT, which is fundamentally involved in regulating lipogenesis and hepatic VLDL secretion [52, 57], ultimately leading to increased HDL clearance [58].

Herein, HFD-induced obese rats treated with the Dapa/IF combination demonstrated normalized body weight and BMI, suggesting an additive effect of their combined administration. Body weight reduction may be ascribed to both Dapa and IF, creating a metabolic state resembling starvation. The nutritional deprivation response seems to activate the p-AMPK/SIRT1 pathway in adipose tissue, as demonstrated in the treated groups. Mechanistically, Dapa and IF activate the p-AMPK/SIRT1 pathway, thereby improving thermogenesis by deacetylating PPARγ and enhancing mitochondrial biogenesis by activating PGC-1α [59, 60]. Furthermore, Dapa induces browning of WAT via activation of the fibroblast growth factor receptor 1 (FGFR1)/liver kinase B1 (LKB1)/AMPK trajectory [61]. Moreover, IF upregulates vascular endothelial growth factor (VEGF), which promotes M2 macrophage activation, increases UCP1 expression, and induces WAT vascularization, all of which are critical for adipose thermogenesis [62].

In our study, the treatment groups showed reduced SIRT7 expression. This finding could be explained by a previous observation linking calorie restriction to SIRT7 degradation [63]. The decline in SIRT7 was associated with decreased adipogenesis, as evidenced by the reduced adiposity index. The most prominent results were observed in the HFD/Dapa + IF group, suggesting a reinforcing effect of Dapa and IF. The more pronounced decline of leptin levels in this group is consistent with previous studies linking low leptin levels to the ability of Dapa and IF to induce fat mass reduction [64, 65]. Beyond diminished adipogenesis, the pronounced elevation of adiponectin in the combination group could be attributable to the effects of Dapa and IF on adiponectin expression by elevating 3-hydroxybutyrate levels [66] and upregulating VEGF expression [62], respectively.

Trendy behaviors have markedly influenced the circadian clock. HFD disrupts the circadian clock persistently and attenuates molecular circadian rhythms [67]. Such a change is associated with disrupted metabolic equilibrium, altered dietary habits, and obesity [68]. The current study revealed increased BMAL1 and CLOCK expression in response to HFD, consistent with an earlier study [69].

The circadian clock is regulated by BMAL1/CLOCK (positive regulators) and CRY/PER proteins (negative regulators). Disruption of the circadian rhythm could be attributed to interactions between sirtuins and clock genes [15]. In our study, the upregulation of BMAL1, CLOCK, and CRY1 in the HFD group was concomitant with SIRT1 downregulation and SIRT7 upregulation. Mechanistically, SIRT1 is directly involved in the BMAL1-CLOCK complex and periodically regulates the acetylation of BMAL1 and histone H3 [15]. Indeed, SIRT1 downregulation disrupts BMAL1 acetylation and, consequently, the formation of the BMAL1-CLOCK complex, leading to subsequent alterations in the circadian cycle [70, 71]. Moreover, SIRT7 overexpression is strongly entangled in circadian clock dysregulation. SIRT7 plays a role in cellular stress by inhibiting the activity of hypoxia-inducible factors such as HIF-1α [15]. HIF-1α stimulates the negative regulators of the circadian rhythm, specifically PER1, PER2, DEC1, and DEC2, thereby suppressing BMAL1/CLOCK heterodimers [72].

A recent study suggests that Dapa modulates circadian rhythm–related pathways, contributing to its cardiometabolic benefits [73]. However, the latter study did not assess the direct effect of Dapa on core circadian clock regulators (BMAL1, CLOCK, CRY1, and SIRT7). Our study provides novel evidence linking Dapa and/or Dapa + IF combination to the regulation of core circadian clock genes in HFD-induced obesity. Interestingly, the combination group showed the largest drop in BMAL1 and CLOCK expression. BMAL1 deletion helps protect against obesity, hyperlipidemia, and fatty liver caused by HFD [74]. This may help explain the greater weight loss seen in the combination group. These findings suggest that lower SIRT7 levels may restore the negative feedback regulation on BMAL1 and CLOCK, ameliorating their gene expression.

In this study, the HFD group showed CRY1 upregulation, consistent with elevated BMAL1/CLOCK expression. Increased CRY1 expression may be attributed to reduced p-AMPK activity, leading to impaired CRY1 degradation and consequent disruption in the negative feedback regulation of circadian signaling [75]. The reduction in CRY1 exerted by Dapa and IF is possibly due to restoring its phosphorylation and degradation by the elevated p-AMPK [75].

Variations in gut microbiota composition may be associated with metabolic disorders [24]. HFD administration resulted in reduced microbial diversity and an elevated Firm/Bac ratio. These results mirror previous reports [76, 77]. A notable prevalence of Firmicutes is associated with exaggerated energy extraction, thereby promoting more calorie absorption and increasing the risk of obesity [78].

Both Dapa and IF can induce a state of starvation, with an anticipated compensatory hyperphagia that typically mitigates weight loss. Surprisingly, this did not occur in our study, potentially due to their combined effect on the modification of the gut microbiome, the regulation of its dynamics, and the modulation of circadian rhythm signaling pathways alongside increased SCFA production. In this study, treatment with Dapa, IF, or both reversed the Firm/Bac ratio, with a more favorable impact of IF or the combination, rescuing the overall changes caused by HFD through increasing gut microbiota richness and diversity. Mechanistically, Bacteroidetes can switch their transcriptional profile to use host-derived glycans when polysaccharides and glycoproteins are unavailable, as in fasting and energy deprivation [79]. Bacteroidetes and Bacteroides may increase in abundance after the depletion of other bacterial groups in response to fasting and energy deprivation, due to their high survival and rapid adaptation to changes in nutrient availability [79, 80]. The Firmicutes phylum decreases in response to elevated Bacteroidetes, aiming to restore a balanced composition and induce structural modifications in the gut microbiota profile resembling that of lean individuals [81]. A recent study showed that Dapa decreased Firm/Bac ratio [82]; however, data on the precise mechanisms by which Dapa modulates Bacteroidetes and Firmicutes remain limited. The present study provides new evidence that Dapa reduces energy availability, potentially leading to a mechanism similar to that induced by IF. Additionally, the correlation between gut microbiome and adipose tissue parameters in our study indicates the potential impact of the gut microbiome on metabolic dysregulation in obesity.

Acetate and propionate are the principal metabolic byproducts of the Bacteroidetes phylum [83]. Notably, acetate and propionate bind GPR43 with high affinity, enhancing POMC expression and promoting the secretion of the anorexigenic PYY, resulting in diminished food intake [84]. Herein, the reduction in acetate and propionate levels observed in the HFD group is likely due to reduced Bacteroidetes. This was accompanied by a notable reduction in serum POMC and PYY, creating an orexigenic environment. Conversely, this group showed GPR43 receptor upregulation, consistent with previous results [85]. Indeed, GPR43-knockout mice exhibited a substantial reduction in body weight and adipose tissue following 16 weeks of HFD exposure [86]. Our results support the hypothesis that receptor expression may be upregulated in response to diminished receptor ligand levels, providing an adaptive mechanism across various physiological contexts [87].

While Dapa selectively elevated serum propionate, IF or the combined Dapa/IF regimen profoundly augmented both acetate and propionate levels back to normal. This finding is concomitant with their more powerful influence on Bacteroidetes abundance than Dapa alone. This was accompanied by elevated anorexigenic peptide levels in the treated groups, more prominently in the combination group. Furthermore, the intervention groups moderated the adaptive mechanism in GPR43, reducing its expression more effectively in the combined regimen. Mechanistically, SCFAs bind to GPR43 and GPR41 receptors, inducing the release of PYY and glucagon-like peptide 1, which activate anorexigenic POMC neurons in the hypothalamic arcuate nucleus [88]. Augmented POMC correlates with the suppression of neuropeptide Y and the enhancement of α-melanocyte-stimulating hormone, which interacts with melanocortin receptors to promote satiety and diminish food consumption [89].

Beyond appetite regulation, the increased SCFAs produced by Dapa and IF may explain their additive effects on energy expenditure, the lessening of hyperglycemia and insulin resistance, and the improvement of the lipid profile. Interestingly, increased acetate may reduce lipid accumulation and promote WAT browning, potentially decreasing body adiposity by enhancing thermogenesis [89]. Propionate reduces hepatic gluconeogenesis by upregulating AMPK [90] and increases the β-cell mass, glucose-stimulated insulin secretion, and glucose uptake through GPR43 activation [91]. Notably, enhanced SCFA production, as demonstrated in the treatment groups, could also elevate adiponectin levels, increasing the insulin sensitivity. Indeed, SCFAs rectified adiponectin's aberrant expression by suppressing DNA methyltransferases and methyl-CpG-binding protein 2, thereby reducing methylation at CpG sites in the adiponectin promoter, and hence its activation [91]. Moreover, increased acetate and propionate have favorable effects on the lipid profile via promoting hepatic cholesterol uptake from the bloodstream, inhibiting the de novo lipogenesis by downregulating hepatic SREBP-1c, FAS, and acetyl-CoA carboxylase-1 expression, and reducing LDL-C levels [92, 93]. Additionally, propionate inhibits triglyceride formation by reducing hepatic triglyceride synthesis and impairing the expression of PPARα-responsive genes [94]. Furthermore, SCFAs enhance hepatic ApoA-I expression, essential for HDL-C production [95]. These multifaceted mechanisms position SCFAs as a significant regulator in obesity management, starting from appetite suppression, induction of weight loss, enhancement of adiponectin production, and modulation of hyperlipidemia.

Regarding butyrate, although butyrate-producing bacteria were detected in most experimental groups, circulating butyrate remained undetectable. This finding is consistent with previous studies demonstrating butyrate as the primary energy substrate for colonocytes and is therefore largely metabolized within the intestinal epithelium [96]. In addition, butyrate undergoes near-complete splanchnic extraction during first-pass metabolism, resulting in minimal release into the peripheral circulation [97]. Consequently, serum butyrate concentrations may remain very low or undetectable despite active colonic production [97, 98]. This highlights an inherent limitation of using circulating butyrate as a surrogate marker of gut microbial fermentation.

In the present study, food and energy intake were quantitatively assessed to determine whether the observed metabolic improvements were attributable to reduced caloric intake or to treatment-specific effects. As expected, IF significantly reduced energy intake compared to the HFD group, reflecting the inherent nature of the fasting regimen rather than a confounding factor. Notably, Dapa did not reduce food or energy intake in HFD-fed rats; instead, the caloric intake in this group was comparable to that of the HFD-alone group. This finding is consistent with the compensatory hyperphagia induced by glucosuria, in line with the drug’s mechanism of action [20–22, 33]. Nonetheless, Dapa administration caused a significant reduction in body weight and metabolic parameters in HFD-induced obese rats, indicating that its beneficial metabolic effects are largely independent of caloric restriction. Importantly, although caloric intake was comparable between the IF and Dapa + IF combination groups, the combined treatment resulted in superior metabolic outcomes, including normalized body weight, improved histopathological and biochemical derangements, modulated expression of p-AMPK/SIRT1, SIRT7, and clock genes, and restored gut microbiome and SCFA levels. This finding suggests a true added benefit by Dapa and IF combination that extends beyond reduced energy intake.

Another outcome of our study is highlighting the beneficial effects of Dapa, IF, or their combination on liver histology and function in HFD-induced obesity. HFD induced marked hepatic injury, as evidenced by significant hepatomegaly, serum ALT and AST elevation, and hepatic fat accumulation. These findings aligns with previous results of HFD-induced hepatic steatosis and liver dysfunction [43, 47]. Treatment with Dapa or IF partially ameliorated these alterations, whereas their combined administration produced more pronounced improvements, as indicated by normalized hepatic architecture, liver size, and enzymes. These findings highlight the superior hepatoprotective effects of the combined intervention over a single treatment, thereby enhancing the translational relevance of our study.

Reversing the detrimental consequences of obesity requires more than just reducing fat accumulation. While IF and Dapa had comparable effects on body weight and adiposity reduction and glycemic control, IF exerted more favorable effects on LDL-C, HDL-C, insulin, leptin, adiponectin, and POMC levels over Dapa in HFD-induced obese rats. These findings might be partly due to the dual elevation of acetate and propionate by IF, whereas Dapa selectively elevated the propionate levels. More interestingly, although the differences in gut microbiome composition and SCFA production between IF monotherapy and the combination treatment were not significant, the Dapa/IF combination group achieved normalized body weight and BMI and a marked reduction in the adiposity index. This highlights the additive effect of Dapa add-on therapy to IF, as reflected in the marked improvement of metabolic and histopathological parameters compared to monotherapy.

This study has several limitations. No age-specific effects were examined, and the interventions were not studied in female animals. The relatively short period of the study precludes drawing conclusions about long-term efficacy or safety. While including the dapa-control group to show the drug safety, the study did not include IF-control and Dapa/IF-control groups as the 16/8 IF model is known with its safety. SIRT1 and SIRT7 were not analyzed at the protein level. Fecal SCFAs were not measured, which could have provided additional insight into gut microbial activity and represent a promising direction for future work. The strict experimental conditions used eliminate the behavioral, environmental, and psychosocial contributors to human weight regulation. In addition, Dapa dosing in rats was optimized and may differ from clinical dose regimens. Thus, it is crucial to emphasize that clinical trials are required to prove the long-term effectiveness of this approach and evaluate individual response variability.

## Conclusion

**Fig. 13:**
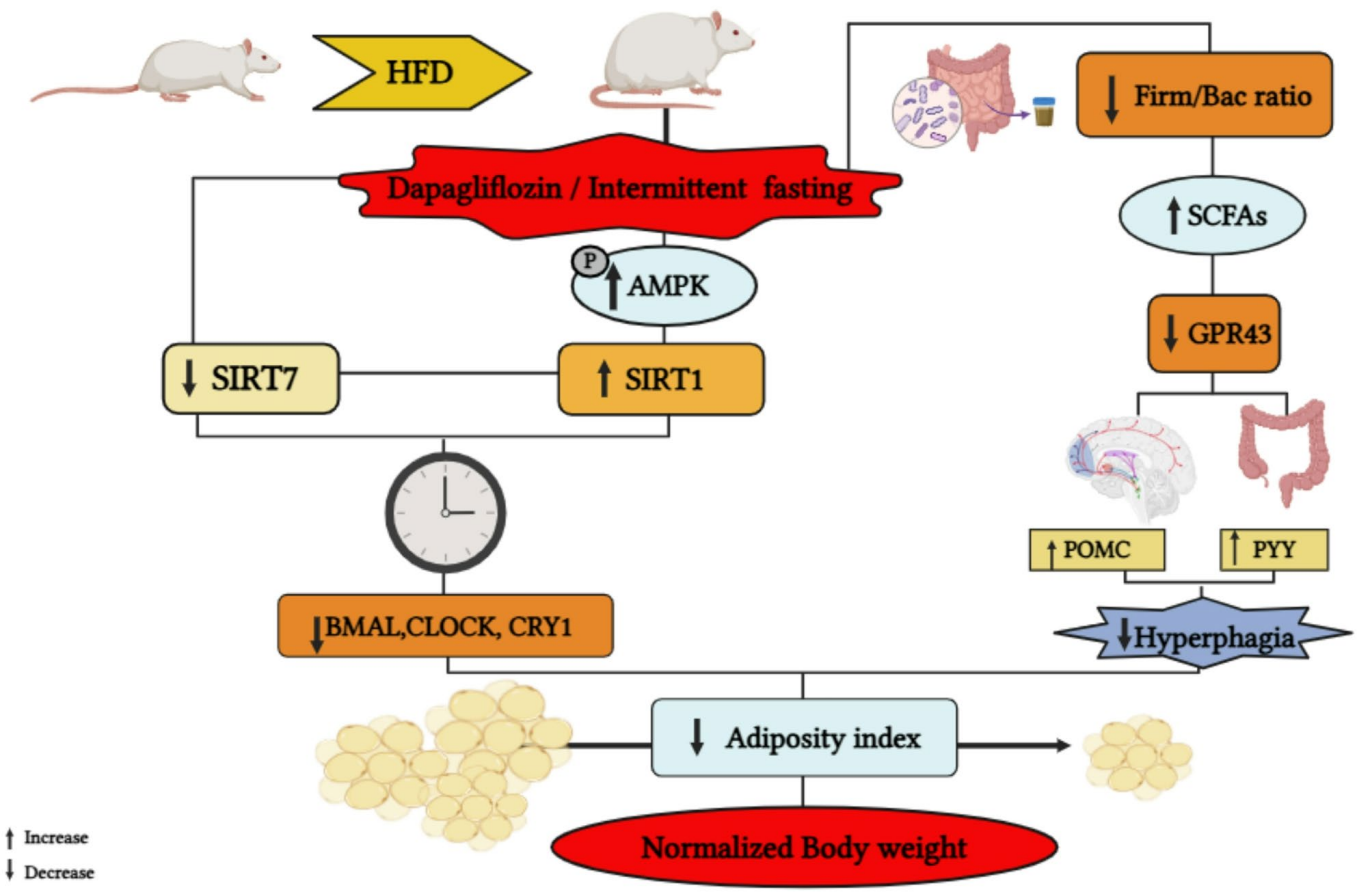
The favorable outcomes of Dapa + IF combination on weight and metabolic regulation through modulating AMPK/Sirtuins/clock genes and restoring gut microbiome signaling

This study is the first to highlight the potential beneficial effects of combined Dapa and IF on experimental obesity treatment and to disclose the impact of Dapa on the expression of key circadian and metabolic markers, such as GPR43, BMAL1, CLOCK, CRY1, and SIRT7, as well as its selectivity towards serum propionate. Combining Dapa and IF can provide extra weight reduction in HFD-induced obesity by augmenting the p-AMPK/SIRT1 axis, mitigating SIRT7, and resetting the clock genes in adipose tissue, while regulating appetite through modulating the gut microbiome to increase SCFA production. These favorable effects resulted in the reduction of adipocyte number, surface area, and fatty deposits, while improving the liver histology and function. Additionally, this combination profoundly improved insulin and leptin resistance, glucose intolerance, and lipid profile derangements over a single treatment. These findings introduce Dapa add-on therapy to IF as an innovative, convenient, and cost-effective approach for facing obesity. A conclusive summary of the main study findings is portrayed in Fig. 13.

## Supplementary Information

Below is the link to the electronic supplementary material.

Supplementary Material 1

## Data Availability

Data are provided within the manuscript and the supplementary file and are available upon request for research purposes.

## References

[1] Guglielmi V, Dalle Grave R, Leonetti F, Solini A. Female obesity: clinical and psychological assessment toward the best treatment. Front Endocrinol. 2024;15:1349794. 10.3389/fendo.2024.1349794. 10.3389/fendo.2024.1349794 PMC11099266 38765954

[2] Okunogbe A, Nugent R, Spencer G, Powis J, Ralston J, Wilding J. Economic impacts of overweight and obesity: current and future estimates for 161 countries. BMJ Glob Health. 2022;7(9):e009773. 10.1136/bmjgh-2022-009773. 36130777 10.1136/bmjgh-2022-009773 PMC9494015

[3] Koliaki C, Dalamaga M, Liatis S. Update on the obesity epidemic: after the sudden rise, is the upward trajectory beginning to flatten? Curr Obes Rep. 2023;12(4):514–27. 10.1007/s13679-023-00527-y. 37779155 10.1007/s13679-023-00527-y PMC10748771

[4] Welsh A, Hammad M, Piña IL, Kulinski J. Obesity and cardiovascular health. Eur J Prev Cardiol. 2024;31(8):1026–35. 10.1093/eurjpc/zwae025. 38243826 10.1093/eurjpc/zwae025 PMC11144464

[5] Trang K, Grant SFA. Genetics and epigenetics in the obesity phenotyping scenario. Rev Endocr Metab Disord. 2023;24(5):775–93. 10.1007/s11154-023-09804-6. 37032403 10.1007/s11154-023-09804-6 PMC10088729

[6] Tsai SF, Hsu PL, Yeh MC, Hung HC, Shih MM, Chung BC, et al. High-fat diet-induced increase in glucocorticoids contributes to adipogenesis in obese mice. Biomed J. 2025;48(3):100772. 10.1016/j.bj.2024.100772. 39048079 10.1016/j.bj.2024.100772 PMC12173011

[7] Ghesmati Z, Rashid M, Fayezi S, Gieseler F, Alizadeh E, Darabi M. An update on the secretory functions of brown, white, and beige adipose tissue: towards therapeutic applications. Rev Endocr Metab Disord. 2024;25(2):279–308. 10.1007/s11154-023-09850-0. 38051471 10.1007/s11154-023-09850-0 PMC10942928

[8] Schirinzi V, Poli C, Berteotti C, Leone A. Browning of adipocytes: a potential therapeutic approach to obesity. Nutrients. 2023;15(9):2229. 10.3390/nu15092229. 37432449 10.3390/nu15092229 PMC10181235

[9] Du Y, Huo Y, Yang Y, Lin P, Liu W, Wang Z, et al. Role of sirtuins in obesity and osteoporosis: molecular mechanisms and therapeutic targets. Cell Commun Signal. 2025;23(1):20. 10.1186/s12964-024-02025-7. 39799353 10.1186/s12964-024-02025-7 PMC11724515

[10] Zhang S, Sun S, Wei X, Zhang M, Chen Y, Mao X, Chen G, Liu C. Short-term moderate caloric restriction in a high-fat diet alleviates obesity via AMPK/SIRT1 signaling in white adipocytes and liver. Food Nutr Res 2022;66. 10.29219/fnr.v66.7909 10.29219/fnr.v66.7909 PMC9180121 35721807

[11] Kurylowicz A, Owczarz M, Polosak J, Jonas MI, Lisik W, Jonas M, et al. SIRT1 and SIRT7 expression in adipose tissues of obese and normal-weight individuals is regulated by microRNAs but not by methylation status. Int J Obes. 2016;40(11):1635–42. 10.1038/ijo.2016.131. 10.1038/ijo.2016.131 27480132

[12] Quan X, Xin Y, Wang HL, Sun Y, Chen C, Zhang J. Implications of altered sirtuins in metabolic regulation and oral cancer. PeerJ. 2023;11:e14752. 10.7717/peerj.14752. 36815979 10.7717/peerj.14752 PMC9936870

[13] Braud L, Pini M, Stec DF, Manin S, Derumeaux G, Stec DE, et al. Increased Sirt1 secreted from visceral white adipose tissue is associated with improved glucose tolerance in obese Nrf2-deficient mice. Redox Biol. 2021;38:101805. 10.1016/j.redox.2020.101805. 33285413 10.1016/j.redox.2020.101805 PMC7721645

[14] Song J, Yang B, Jia X, Li M, Tan W, Ma S, et al. Distinctive roles of sirtuins on diabetes, protective or detrimental? Front Endocrinol. 2018;9:724. 10.3389/fendo.2018.00724. 10.3389/fendo.2018.00724 PMC6284472 30559718

[15] Zhuang Y, Zhang Y, Liu C, Zhong Y. Interplay between the circadian clock and sirtuins. Int J Mol Sci. 2024;25(21):11469. 10.3390/ijms252111469. 39519022 10.3390/ijms252111469 PMC11545976

[16] Pilorz V, Helfrich-Förster C, Oster H. The role of the circadian clock system in physiology. Pflugers Arch - Eur J Physiol. 2018;470(2):227–39. 10.1007/s00424-017-2103-y. 29302752 10.1007/s00424-017-2103-y

[17] Zanquetta MM, Corrêa-Giannella ML, Monteiro MB, Villares SM. Body weight, metabolism and clock genes. Diabetol Metab Syndr. 2010;2:53. 10.1186/1758-5996-2-53. 20712885 10.1186/1758-5996-2-53 PMC2930623

[18] Liao J, Yin Y, Zhong J, Chen Y, Chen Y, Wen Y, et al. Bariatric surgery and health outcomes: an umbrella analysis. Front Endocrinol. 2022;13:1016613. 10.3389/fendo.2022.1016613. 10.3389/fendo.2022.1016613 PMC9650489 36387921

[19] Jaikumkao K, Thongnak L, Htun KT, Pengrattanachot N, Phengpol N, Sutthasupha P, et al. Dapagliflozin and metformin in combination ameliorates diabetic nephropathy by suppressing oxidative stress, inflammation, and apoptosis and activating autophagy in diabetic rats. Biochimica et biophysica acta. Mol Basis Dis. 2024;1870(1):166912. 10.1016/j.bbadis.2023.166912. 10.1016/j.bbadis.2023.166912 37816397

[20] Horie I, Abiru N, Hongo R, Nakamura T, Ito A, Haraguchi A, et al. Increased sugar intake as a form of compensatory hyperphagia in patients with type 2 diabetes under dapagliflozin treatment. Diabetes Res Clin Pract. 2018;135:178–84. 10.1016/j.diabres.2017.11.016. 29162514 10.1016/j.diabres.2017.11.016

[21] Chiba Y, Yamada T, Tsukita S, Takahashi K, Munakata Y, Shirai Y, et al. Dapagliflozin, a Sodium-Glucose Co-Transporter 2 inhibitor, acutely reduces energy expenditure in BAT via neural signals in mice. PLoS ONE. 2016;11(3):e0150756. 10.1371/journal.pone.0150756. 26963613 10.1371/journal.pone.0150756 PMC4786146

[22] Gao Q, Jiang Y, Song Z, Ren H, Kong Y, Wang C, et al. Dapagliflozin improves skeletal muscle insulin sensitivity through SIRT1 activation induced by nutrient deprivation state. Sci Rep. 2024;14(1):16878. 10.1038/s41598-024-67755-7. 39043740 10.1038/s41598-024-67755-7 PMC11266597

[23] Yang M, Shi FH, Liu W, Zhang MC, Feng RL, Qian C, et al. Dapagliflozin Modulates the Fecal Microbiota in a Type 2 Diabetic Rat Model. Front Endocrinol (Lausanne). 2020;11:635. 10.3389/fendo.2020.00635. 10.3389/fendo.2020.00635 PMC7707060 33312157

[24] Afzaal M, Saeed F, Shah YA, Hussain M, Rabail R, Socol CT, et al. Human gut microbiota in health and disease: unveiling the relationship. Front Microbiol. 2022;13:999001. 10.3389/fmicb.2022.999001. 36225386 10.3389/fmicb.2022.999001 PMC9549250

[25] Senghor B, Sokhna C, Ruimy R, Lagier J. Gut microbiota diversity according to dietary habits and geographical provenance. Hum Microbiome J. 2018;7–8:1–9. 10.1016/j.humic.2018.01.001.

[26] Nogal A, Valdes AM, Menni C. The role of short-chain fatty acids in the interplay between gut microbiota and diet in cardio-metabolic health. Gut Microbes. 2021;13(1):1–24. 10.1080/19490976.2021.1897212. 33764858 10.1080/19490976.2021.1897212 PMC8007165

[27] Facchin S, Bertin L, Bonazzi E, Lorenzon G, De Barba C, Barberio B, et al. Short-chain fatty acids and human health: from metabolic pathways to current therapeutic implications. Life. 2024;14(5):559. 10.3390/life14050559. 38792581 10.3390/life14050559 PMC11122327

[28] Bahadoran Z, Mirmiran P, Kashfi K, Ghasemi A. Effects of time-restricted feeding (TRF)-model of intermittent fasting on adipose organ: a narrative review. Eat Weight Disord. 2024;29(1):77. 10.1007/s40519-024-01709-w. 39719521 10.1007/s40519-024-01709-w PMC11668836

[29] Xiaoyu W, Yuxin X, Li L. The effects of different intermittent fasting regimens in people with type 2 diabetes: a network meta-analysis. Front Nutr. 2024;11:1325894. 10.3389/fnut.2024.1325894. 38332802 10.3389/fnut.2024.1325894 PMC10850351

[30] García-Gaytán AC, Miranda-Anaya M, Turrubiate I, López-De Portugal L, Bocanegra-Botello GN, López-Islas A, et al. Synchronization of the circadian clock by time-restricted feeding with progressive increasing calorie intake. Resemblances and differences regarding a sustained hypocaloric restriction. Sci Rep. 2020;10(1):10036. 10.1038/s41598-020-66538-0. 32572063 10.1038/s41598-020-66538-0 PMC7308331

[31] Su J, Wang Y, Zhang X, Ma M, Xie Z, Pan Q, et al. Remodeling of the gut microbiome during Ramadan-associated intermittent fasting. Am J Clin Nutr. 2021;113(5):1332–42. 10.1093/ajcn/nqaa388. 33842951 10.1093/ajcn/nqaa388 PMC8106760

[32] Loh MY, Flatt WP, Martin RJ, Hausman DB. Dietary fat type and level influence adiposity development in obese but not lean Zucker rats. Proc Soc Exp Biol Med. 1998;218(1):38–44. 10.3181/00379727-218-44265. 9572150 10.3181/00379727-218-44265

[33] - Devenny JJ, Godonis HE, Harvey SJ, Rooney S, Cullen MJ, Pelleymounter MA. Weight loss induced by chronic dapagliflozin treatment is attenuated by compensatory hyperphagia in diet-induced obese (DIO) rats. Obesity(Silver Spring). 2012;20(8):1645–1652. 10.1038/oby.2012.59. 10.1038/oby.2012.59 22402735

[34] Gerbaix M, Metz L, Ringot E, Courteix D. Visceral fat mass determination in rodent: validation of dual-energy x-ray absorptiometry and anthropometric techniques in fat and lean rats. Lipids Health Dis. 2010;9(1):140. 10.1186/1476-511x-9-140. 21143884 10.1186/1476-511X-9-140 PMC3014952

[35] Novelli EL, Diniz YS, Galhardi CM, et al. Anthropometrical parameters and markers of obesity in rats. Lab Anim. 2007;41(1):111–9. 10.1258/002367707779399518. 17234057 10.1258/002367707779399518

[36] Bradford MM. A rapid and sensitive method for the quantitation of microgram quantities of protein utilizing the principle of protein-dye binding. Anal Biochem. 1976;72:248–54. 942051 10.1016/0003-2697(76)90527-3

[37] Folch J, Lees M, Sloane Stanley GH. A simple method for the isolation and purification of total lipides from animal tissues. J Biol Chem. 1957;226(1):497–509. 13428781

[38] Preda A, Montecucco F, Carbone F, Camici GG, Lüscher TF, Kraler S, et al. SGLT2 inhibitors: from glucose-lowering to cardiovascular benefits. Cardiovasc Res. 2024;120(5):443–60. 10.1093/cvr/cvae047. 38456601 10.1093/cvr/cvae047 PMC12001887

[39] Bhoumik S, Kumar R, Rizvi SI. Time restricted feeding provides a viable alternative to alternate day fasting when evaluated in terms of redox homeostasis in rats. Arch Gerontol Geriatr. 2020;91:104188. 10.1016/j.archger.2020.104188. 32717588 10.1016/j.archger.2020.104188

[40] Doulberis M, Papaefthymiou A, Polyzos SA, Katsinelos P, Grigoriadis N, Srivastava DS, et al. Rodent models of obesity. Minerva Endocrinol. 2020;45(3):243–63. 10.23736/S0391-1977.19.03058-X. 31738033 10.23736/S0391-1977.19.03058-X

[41] Yu R, Yuan J, Ma L, Qin Q, Wu X. Probiotics improve obesity-associated dyslipidemia and insulin resistance in high-fat diet-fed rats. PubMed. 2013;15(12):1123–7. 24342213

[42] Yousef HN, Saleh AA. Lipid profile and some hormonal disorders in serum of High-Fat diet fed rats. Egypt J Hosp Med. 2013;52:615–23. 10.12816/0000598.

[43] Yuan Y, Liu Q, Zhao F, Cao J, Shen X, Li C. Holothuria leucospilota Polysaccharides ameliorate hyperlipidemia in High-Fat Diet-Induced rats via Short-Chain fatty acids production and lipid metabolism regulation. Int J Mol Sci. 2019;20(19):4738. 10.3390/ijms20194738. 31554265 10.3390/ijms20194738 PMC6801986

[44] Bernecker M, Lin A, Feuchtinger A, Molenaar A, Schriever SC, Pfluger PT. Weight cycling exacerbates glucose intolerance and hepatic triglyceride storage in mice with a history of chronic high fat diet exposure. J Transl Med. 2025;23(1):7. 10.1186/s12967-024-06039-0. 39754229 10.1186/s12967-024-06039-0 PMC11699648

[45] Eom J, Thomas SS, Sung NY, Kim DS, Cha YS, Kim KA. Abeliophyllum distichum ameliorates high-fat diet-induced obesity in C57BL/6J mice by upregulating the AMPK pathway. Nutrients. 2020;12(11):3320. 10.3390/nu12113320. 33138026 10.3390/nu12113320 PMC7692136

[46] Xu C, Zhang X, Wang Y, Wang Y, Zhou Y, Li F, et al. Dietary kaempferol exerts anti-obesity effects by inducing the browing of white adipocytes via the AMPK/SIRT1/PGC-1α signaling pathway. Curr Res Food Sci. 2024;8:100728. 10.1016/j.crfs.2024.100728. 38577419 10.1016/j.crfs.2024.100728 PMC10990952

[47] Zhang N, Liu T, Wang J, Xiao Y, Zhang Y, Dai J, et al. Si-Ni-San reduces hepatic lipid deposition in rats with metabolic associated fatty liver disease by AMPK/SIRT1 pathway. Drug Des Devel Ther. 2023;17:3047–60. 10.2147/DDDT.S417378. 37808345 10.2147/DDDT.S417378 PMC10559901

[48] Mayoral R, Osborn O, McNelis J, Johnson AM, Oh DY, Izquierdo CL, et al. Adipocyte SIRT1 knockout promotes PPARγ activity, adipogenesis and insulin sensitivity in chronic-HFD and obesity. Mol Metab. 2015;4(5):378–91. 10.1016/j.molmet.2015.02.007. 25973386 10.1016/j.molmet.2015.02.007 PMC4421024

[49] Yong-Ru C, Yu-lin L, Shaoda L, Xitao L, Yu-Cai F, Wencan X. SIRT1 interacts with metabolic transcriptional factors in the pancreas of insulin-resistant and calorie-restricted rats. Mol Biol Rep. 2013;40(4):3373–80. 10.1007/S11033-012-2412-3. 23292098 10.1007/s11033-012-2412-3

[50] Yoshizaki T, Milne JC, Imamura T, Schenk S, Sonoda N, Babendure JL, et al. SIRT1 exerts anti-inflammatory effects and improves insulin sensitivity in adipocytes. Mol Cell Biol. 2009;29(5):1363–74. 10.1128/MCB.00705-08. 19103747 10.1128/MCB.00705-08 PMC2643824

[51] Iwabu M, Okada-Iwabu M, Yamauchi T, Kadowaki T. Adiponectin/AdipoR research and its implications for lifestyle-related diseases. Front Cardiovasc Med. 2019;6:116. 10.3389/fcvm.2019.00116. 31475160 10.3389/fcvm.2019.00116 PMC6703139

[52] Zhou S, Tang X, Chen H-Z. Sirtuins and insulin resistance. Front Endocrinol. 2018;9:748-748. 10.3389/FENDO.2018.00748. 10.3389/fendo.2018.00748 PMC6291425 30574122

[53] Akter F, Tsuyama T, Yoshizawa T, Sobuz SU, Yamagata K. SIRT7 regulates lipogenesis in adipocytes through deacetylation of PPARγ2. J Diabetes Investig. 2021;12(10):1765–74. 10.1111/jdi.13567. 33955199 10.1111/jdi.13567 PMC8504911

[54] Kirichenko TV, Markina YV, Bogatyreva AI, Tolstik TV, Varaeva YR, Starodubova AV. The role of adipokines in inflammatory mechanisms of obesity. Int J Mol Sci. 2022;23(23):14982. 10.3390/ijms232314982. 36499312 10.3390/ijms232314982 PMC9740598

[55] Münzberg H, Heymsfield SB. New insights into the regulation of Leptin gene expression. Cell Metab. 2019;29(5):1013–4. 10.1016/j.cmet.2019.04.005. 31067443 10.1016/j.cmet.2019.04.005 PMC7346278

[56] Andrés-Blasco I, Blesa S, Vinué Á, González-Navarro H, Real JT, Martínez-Hervás S, et al. Srebf2 locus overexpression reduces body weight, total cholesterol and glucose levels in mice fed with two different diets. Nutrients. 2020;12(10):3130. 10.3390/nu12103130. 33066385 10.3390/nu12103130 PMC7602228

[57] Rada P, González-Rodríguez Á, García-Monzón C, Valverde ÁM. Understanding lipotoxicity in NAFLD pathogenesis: is CD36 a key driver? Cell Death Dis. 2020;11(9):802. 10.1038/s41419-020-03003-w. 32978374 10.1038/s41419-020-03003-w PMC7519685

[58] Wang H, Peng DQ. New insights into the mechanism of low high-density lipoprotein cholesterol in obesity. Lipids Health Dis. 2011;10:176. 10.1186/1476-511X-10-176. 21988829 10.1186/1476-511X-10-176 PMC3207906

[59] Faridvand Y, Kazemzadeh H, Vahedian V, Mirzajanzadeh P, Nejabati HR, Safaie N, et al. Dapagliflozin attenuates high glucose-induced endothelial cell apoptosis and inflammation through AMPK/SIRT1 activation. Clin Exp Pharmacol Physiol. 2022;49(6):643–51. 10.1111/1440-1681.13638. 35274762 10.1111/1440-1681.13638

[60] Yuliyanasari N, Rejeki PS, Hidayati HB, Subsomwong P, Miftahussurur M. The effect of intermittent fasting on preventing obesity-related early aging from a molecular and cellular perspective. J Med Life. 2024;17(3):261–72. 10.25122/jml-2023-0370. 39044934 10.25122/jml-2023-0370 PMC11262604

[61] Lv Y, Zhao C, Jiang Q, Rong Y, Ma M, Liang L, et al. Dapagliflozin promotes browning of white adipose tissue through the FGFR1-LKB1-AMPK signaling pathway. Mol Biol Rep. 2024;51(1):562. 10.1007/s11033-024-09540-3. 38644407 10.1007/s11033-024-09540-3 PMC11033239

[62] Kim K, Kim YH, Son JE, Lee JH, Kim S, Choe MS, et al. Intermittent fasting promotes adipose thermogenesis and metabolic homeostasis via VEGF-mediated alternative activation of macrophage. Cell Res. 2017;27(11):1309–26. 10.1038/cr.2017.126. 29039412 10.1038/cr.2017.126 PMC5674160

[63] Tang X, Li G, Shi L, Su F, Qian M, Liu Z, et al. Combined intermittent fasting and ERK inhibition enhance the anti-tumor effects of chemotherapy via the GSK3β-SIRT7 axis. Nat Commun. 2021;12(1):5058. 10.1038/s41467-021-25274-3. 34433808 10.1038/s41467-021-25274-3 PMC8387475

[64] Chen X, Xiao Z, Dai N, Fan M. Impact of dapagliflozin on metabolic phenotype, hormone levels, and fertility in female mice after prolonged high-fat diet. Front Endocrinol (Lausanne). 2025;15:1457268. 10.3389/fendo.2024.1457268. 10.3389/fendo.2024.1457268 PMC11791800 39906039

[65] Mushtaq R, Akram A, Mushtaq R, Ahmed S. Effect of Ramadan fasting on body weight and serum leptin level: a prospective study. J Dow Univ Health Sci. 2019;13(1):3–9. 10.36570/jduhs.2019.1.586.

[66] Nishitani S, Fukuhara A, Shin J, Okuno Y, Otsuki M, Shimomura I. Metabolomic and microarray analyses of adipose tissue of dapagliflozin-treated mice, and effects of 3-hydroxybutyrate on induction of adiponectin in adipocytes. Sci Rep. 2018;8(1):8805. 10.1038/s41598-018-27181-y. 29891844 10.1038/s41598-018-27181-y PMC5995811

[67] Hatori M, Vollmers C, Zarrinpar A, DiTacchio L, Bushong EA, Gill S, et al. Time-restricted feeding without reducing caloric intake prevents metabolic diseases in mice fed a high-fat diet. Cell Metab. 2012;15(6):848–60. 10.1016/j.cmet.2012.04.019. 22608008 10.1016/j.cmet.2012.04.019 PMC3491655

[68] Wójcik M, Alvarez-Pitti J, Kozioł-Kozakowska A, Brzeziński M, Gabbianelli R, Herceg-Čavrak V, et al. Psychosocial and environmental risk factors of obesity and hypertension in children and adolescents-a literature overview. Front Cardiovasc Med. 2023;10:1268364. 10.3389/fcvm.2023.1268364. 38054100 10.3389/fcvm.2023.1268364 PMC10694215

[69] Vieira E, Ruano E, Figueroa AL, Aranda G, Momblan D, Carmona F, et al. Altered clock gene expression in obese visceral adipose tissue is associated with metabolic syndrome. PLoS ONE. 2014;9(11):e111678. 10.1371/journal.pone.0111678. 25365257 10.1371/journal.pone.0111678 PMC4218799

[70] Nakahata Y, Kaluzova M, Grimaldi B, Sahar S, Hirayama J, Chen D, et al. The NAD+-dependent deacetylase SIRT1 modulates CLOCK-mediated chromatin remodeling and circadian control. Cell. 2008;134(2):329–40. 10.1016/j.cell.2008.07.002. 18662547 10.1016/j.cell.2008.07.002 PMC3526943

[71] Tong X, Zhang D, Arthurs B, Li P, Durudogan L, Gupta N, et al. Palmitate inhibits SIRT1-dependent BMAL1/CLOCK interaction and disrupts circadian gene oscillations in hepatocytes. PLoS ONE. 2015;10(6):e0130047. 10.1371/journal.pone.0130047. 26075729 10.1371/journal.pone.0130047 PMC4468094

[72] Juhász KZ, Hajdú T, Kovács P, Vágó J, Matta C, Takács R. Hypoxic conditions modulate chondrogenesis through the circadian clock: the role of Hypoxia-Inducible Factor-1α. Cells. 2024;13(6):512. 10.3390/cells13060512. 38534356 10.3390/cells13060512 PMC10969332

[73] Wang J, She Q, Du J. Dapagliflozin attenuates myocardial remodeling in hypertension by activating the circadian rhythm signaling pathway. Arch Pharm Res. 2023;46(2):117–30. 10.1007/s12272-023-01430-9. 36729273 10.1007/s12272-023-01430-9

[74] Zhan C, Chen H, Zhang Z, Shao Y, Xu B, Hua R, et al. BMAL1 deletion protects against obesity and non-alcoholic fatty liver disease induced by a high-fat diet. Int J Obes. 2024;48(4):469–76. 10.1038/s41366-023-01435-w. 10.1038/s41366-023-01435-w 38081925

[75] Qiu J, Dai T, Tao H, Li X, Luo C, Sima Y, et al. Inhibition of expression of the circadian clock gene Cryptochrome 1 causes abnormal glucometabolic and cell growth in *Bombyx mori* cells. Int J Mol Sci. 2023;24(6):5435. 10.3390/ijms24065435. 36982509 10.3390/ijms24065435 PMC10056408

[76] Rosenbaum M, Knight R, Leibel RL. The gut microbiota in human energy homeostasis and obesity. Trends Endocrinol Metab. 2015;26(9):493–501. 10.1016/j.tem.2015.07.002. 26257300 10.1016/j.tem.2015.07.002 PMC4862197

[77] Petakh P, Oksenych V, Kamyshnyi A. The F/B ratio as a biomarker for inflammation in COVID-19 and T2D: impact of metformin. Biomed Pharmacother. 2023;163:114892. 10.1016/j.biopha.2023.114892. 37196542 10.1016/j.biopha.2023.114892 PMC10183625

[78] Krajmalnik-Brown R, Ilhan ZE, Kang DW, DiBaise JK. Effects of gut microbes on nutrient absorption and energy regulation. Nutr Clin Pract. 2012;27(2):201–14. 10.1177/0884533611436116. 22367888 10.1177/0884533611436116 PMC3601187

[79] Brown HA, Koropatkin NM. Host glycan utilization within the Bacteroidetes Sus-like paradigm. Glycobiology. 2021;31(6):697–706. 10.1093/glycob/cwaa054. 32518945 10.1093/glycob/cwaa054 PMC8252860

[80] Özkul C, Yalınay M, Karakan T. Islamic fasting leads to an increased abundance of *Akkermansia muciniphila* and *Bacteroides fragilis* group: a preliminary study on intermittent fasting. Turk J Gastroenterol. 2019;30(12):1030–5. 10.5152/tjg.2019.19185. 31854308 10.5152/tjg.2019.19185 PMC6924600

[81] Zeng T, Cui H, Tang D, Garside GB, Wang Y, Wu J, et al. Short-term dietary restriction in old mice rejuvenates the aging-induced structural imbalance of gut microbiota. Biogerontology. 2019;20(6):837–48. 10.1007/s10522-019-09830-5. 31401701 10.1007/s10522-019-09830-5 PMC6790194

[82] Wang L, Wang Y, Xu H, Li W. Effect of dapagliflozin on ferroptosis through the gut microbiota metabolite TMAO during myocardial ischemia-reperfusion injury in diabetes mellitus rats. Sci Rep. 2024;14(1):13851. 10.1038/s41598-024-64909-5. 38879701 10.1038/s41598-024-64909-5 PMC11180094

[83] Parada Venegas D, De la Fuente MK, Landskron G, González MJ, Quera R, Dijkstra G, et al. Short chain fatty acids (SCFAs)-mediated gut epithelial and immune regulation and its relevance for inflammatory bowel diseases. Front Immunol. 2019;10:277. 10.3389/fimmu.2019.00277. 30915065 10.3389/fimmu.2019.00277 PMC6421268

[84] Yu M, Yu B, Chen D. The effects of gut microbiota on appetite regulation and the underlying mechanisms. Gut Microbes. 2024;16(1):2414796. 10.1080/19490976.2024.2414796. 39501848 10.1080/19490976.2024.2414796 PMC11542600

[85] Hong YH, Nishimura Y, Hishikawa D, Tsuzuki H, Miyahara H, Gotoh C, et al. Acetate and propionate short chain fatty acids stimulate adipogenesis via GPCR43. Endocrinology. 2005;146(12):5092–9. 10.1210/en.2005-0545. 16123168 10.1210/en.2005-0545

[86] Bjursell M, Admyre T, Göransson M, Marley AE, Smith DM, Oscarsson J, et al. Improved glucose control and reduced body fat mass in free fatty acid receptor 2-deficient mice fed a high-fat diet. Am J Physiol Endocrinol Metab. 2011;300(1):E211–20. 10.1152/ajpendo.00229.2010. 20959533 10.1152/ajpendo.00229.2010

[87] Bittner EA, Martyn J. ‘21-neuromuscular physiology and pharmacology,”. In: Hemmings HC, Egan TD, editors. Pharmacology and physiology for anesthesia. 2nd ed. Philadelphia: Elsevier; 2019. p. 412–27. 10.1016/B978-0-323-48110-6.00021-1.

[88] De Silva A, Bloom SR. Gut hormones and appetite control: a focus on PYY and GLP-1 as therapeutic targets in obesity. Gut Liver. 2012;6(1):10–20. 10.5009/gnl.2012.6.1.10. 22375166 10.5009/gnl.2012.6.1.10 PMC3286726

[89] Sahuri-Arisoylu M, Brody LP, Parkinson JR, Parkes H, Navaratnam N, Miller AD, et al. Reprogramming of hepatic fat accumulation and “browning” of adipose tissue by the short-chain fatty acid acetate. Int J Obes (Lond). 2016;40(6):955–63. 10.1038/ijo.2016.23. 26975441 10.1038/ijo.2016.23

[90] Yoshida H, Ishii M, Akagawa M. Propionate suppresses hepatic gluconeogenesis via GPR43/AMPK signaling pathway. Arch Biochem Biophys. 2019;672:108057. 10.1016/j.abb.2019.07.022. 31356781 10.1016/j.abb.2019.07.022

[91] Lu Y, Fan C, Liang A, Fan X, Wang R, Li P, et al. Effects of SCFA on the DNA methylation pattern of adiponectin and resistin in high-fat-diet-induced obese male mice. Br J Nutr. 2018;120(4):385–92. 10.1017/S0007114518001526. 29925443 10.1017/S0007114518001526

[92] Shah S, Fillier T, Pham TH, Thomas R, Cheema SK. Intraperitoneal administration of short-chain fatty acids improves lipid metabolism of long-Evans rats in a sex-specific manner. Nutrients. 2021;13(3):892. 10.3390/nu13030892. 33801984 10.3390/nu13030892 PMC8000072

[93] Zhao Y, Liu J, Hao W, Zhu H, Liang N, He Z, et al. Structure-specific effects of short-chain fatty acids on plasma cholesterol concentration in Male Syrian Hamsters. J Agric Food Chem. 2017;65(50):10984–92. 10.1021/acs.jafc.7b04666. 29190422 10.1021/acs.jafc.7b04666

[94] Cook KJ, Coulter A, Keenan M, Greenway F, Losso JN. Sodium propionate or sodium butyrate promotes fatty acid oxidation in HepG2 cells under oxidative stress. J Med Food. 2023;26(1):74–9. 10.1089/jmf.2021.0120. 36637439 10.1089/jmf.2021.0120 PMC9889010

[95] Tayyeb JZ, Popeijus HE, Mensink RP, Konings MCJM, Mulders KHR, Plat J. The effects of short-chain fatty acids on the transcription and secretion of apolipoprotein A-I in human hepatocytes in vitro. J Cell Biochem. 2019;120(10):17219–27. 10.1002/jcb.28982. 31106471 10.1002/jcb.28982 PMC6767783

[96] Boets E, Gomand SV, Deroover L, Preston T, Vermeulen K, De Preter V, et al. Systemic availability and metabolism of colonic-derived short-chain fatty acids in healthy subjects: a stable isotope study. J Physiol. 2017;595(2):541–55. 10.1113/JP272613. 27510655 10.1113/JP272613 PMC5233652

[97] Ozkul C, Yalinay M, Karakan T. Structural changes in gut microbiome after Ramadan fasting: a pilot study. Benef Microbes. 2020;11(3):227–33. 10.3920/BM2019.0039. 32073296 10.3920/BM2019.0039

[98] Cignarella F, Cantoni C, Ghezzi L, Salter A, Dorsett Y, Chen L, et al. Intermittent fasting confers protection in CNS autoimmunity by altering the gut microbiota. Cell Metab. 2018;27(6):1222-1235.e6. 10.1016/j.cmet.2018.05.006. 29874567 10.1016/j.cmet.2018.05.006 PMC6460288

