# Supplementary material for: Dapagliflozin-intermittent fasting combination maximizes weight and metabolic regulation through AMPK/sirtuins/clock genes and gut microbiota signaling in high-fat diet-induced obesity: a novel anti-obesity approach
Source: Cell Biosci. 2026 Apr 12;16:41. doi: 10.1186/s13578-026-01557-4 (PMC13088541; doi:10.1186/s13578-026-01557-4)
Supplement: Supplementary file 1 — Supplementary Material 1 [file 13578_2026_1557_MOESM1_ESM.docx]

**Supplementary Information**


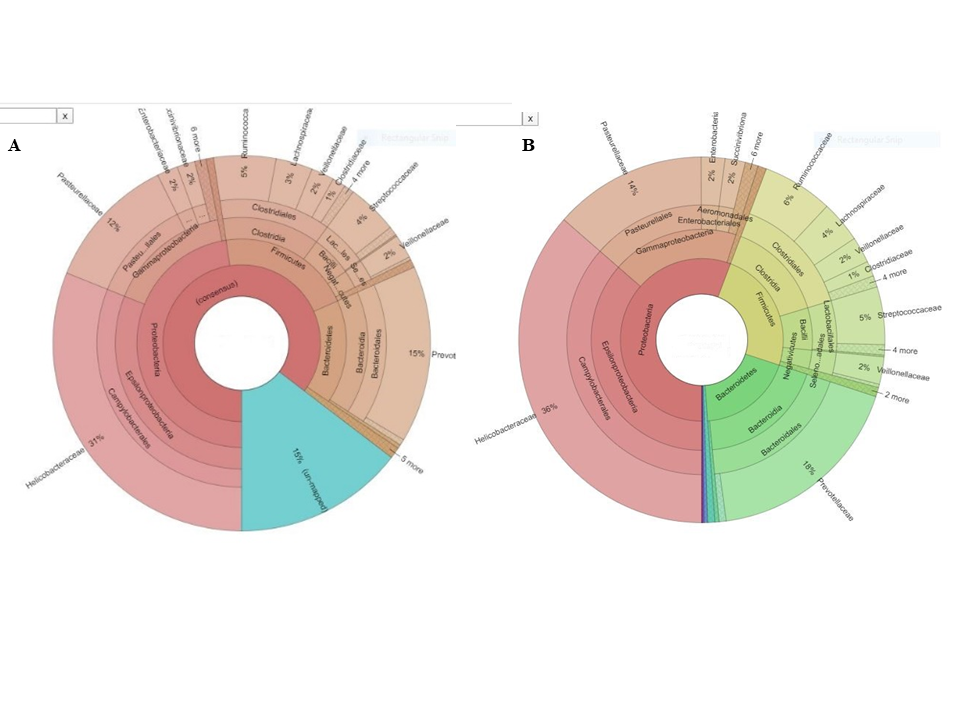


***Figure S1. The Krona charts showing the relative abundance of the gut microbiome at the class, order, genus levels in rats’ intestinal content in different studied groups:*** *A) control, B) Dapa.*


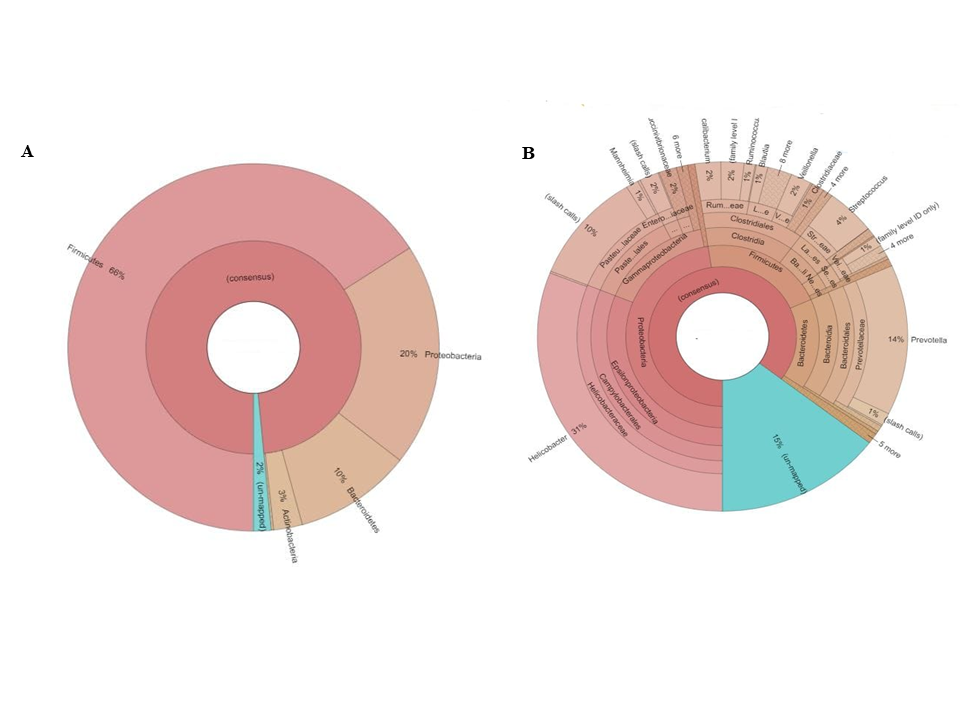


***Figure S2. The Krona charts showing the relative abundance of the gut microbiome at the class, order, genus levels in rats’ intestinal content in different studied groups:***  *A) HFD, B) HFD/Dapa.*


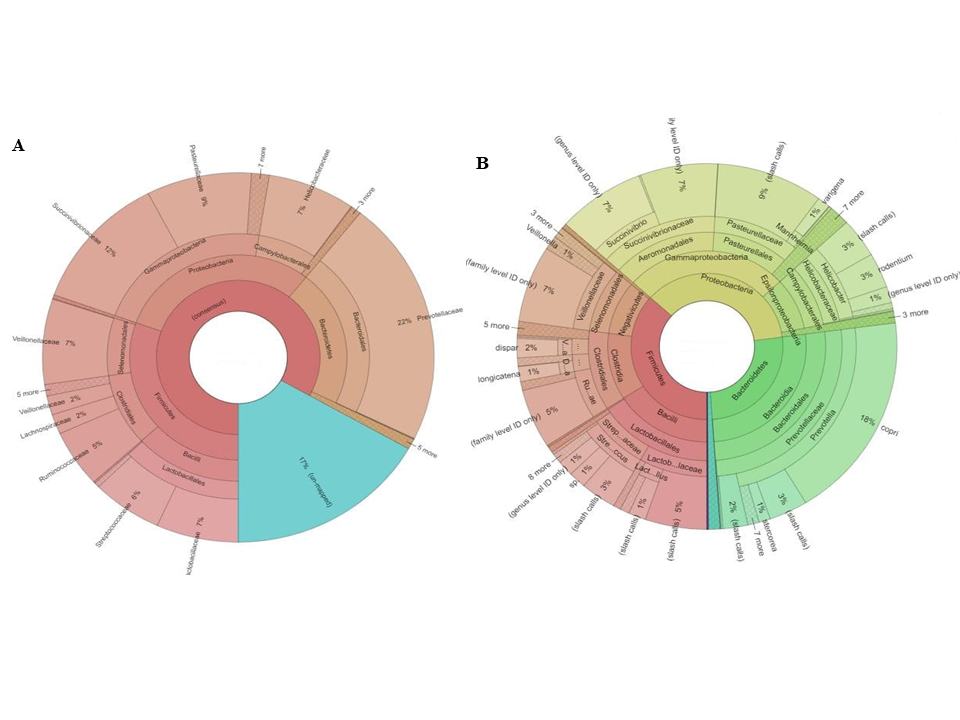


***Figure S3. The Krona charts showing the relative abundance of the gut microbiome at the class, order, genus levels in rats’ intestinal content in different studied groups:*** *A) HFD/IF, B) HFD/Dapa+IF.*

***
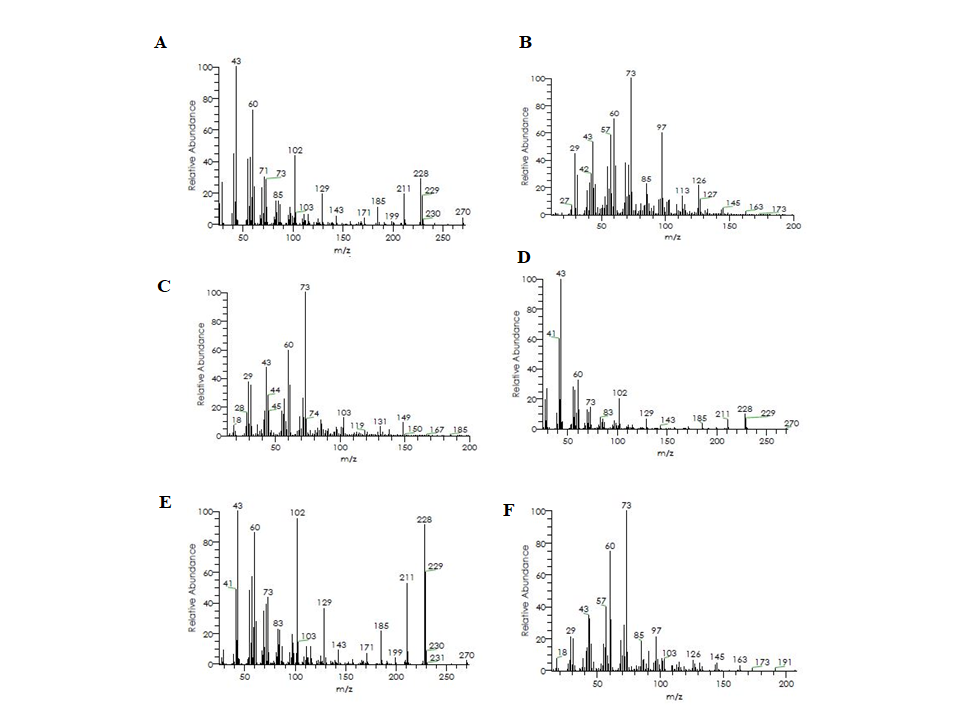
***

***Figure S4. Mass spectra of serum acetate in HFD-induced obesity in rats.*** *A: control, B: Dapa, C: HFD, D: HFD/Dapa, E: HFD/IF, F: HFD/Dapa+IF .*

***
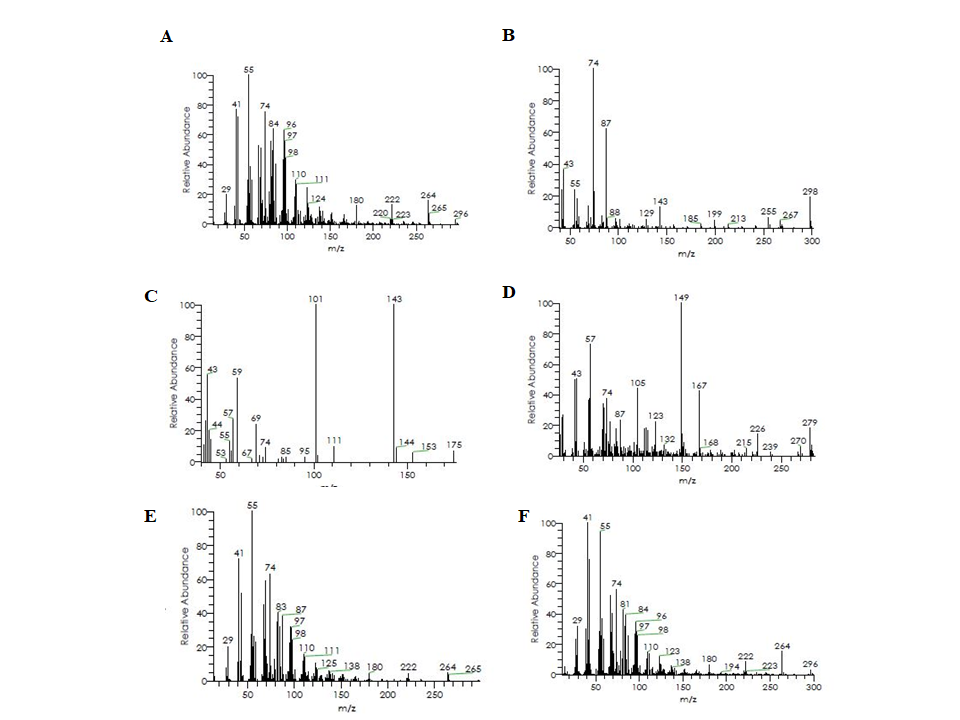
***

***Figure S5. Mass spectra of serum propionate in HFD-induced obesity in rats.*** *A: control, B: Dapa, C: HFD, D: HFD/Dapa, E: HFD/IF, F: HFD/Dapa+IF.*


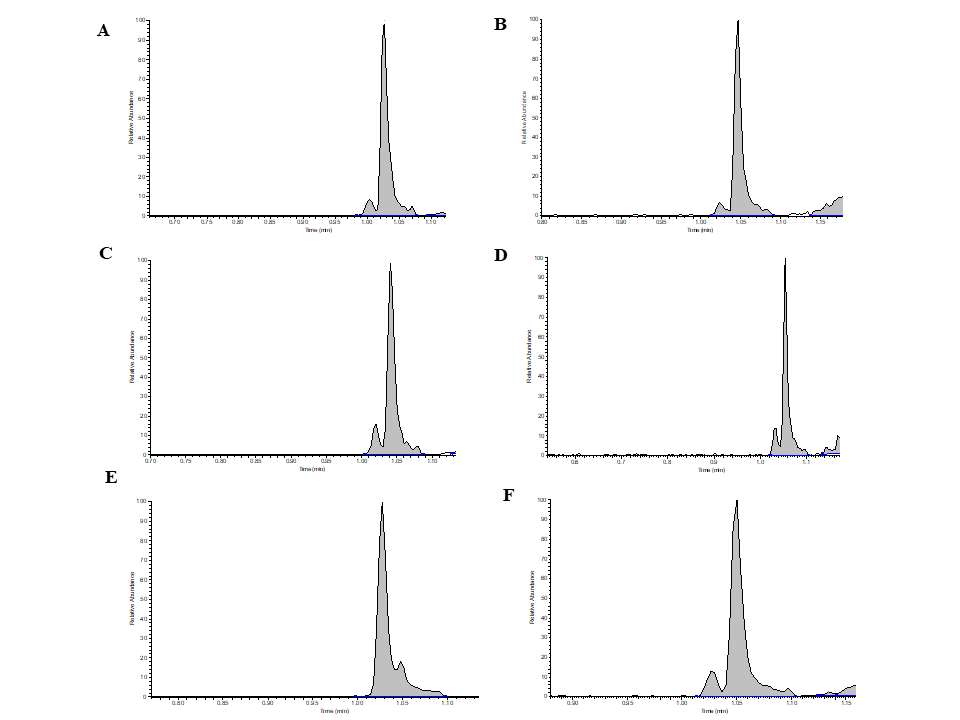


***Figure S6. Representative chromatograms for serum acetate in HFD-induced obesity in rats.*** *A: control, B: Dapa, C: HFD, D: HFD/Dapa, E: HFD/IF, F: HFD/Dapa+IF. The y-axis represents relative abundance and the x-axis represents time (min).*


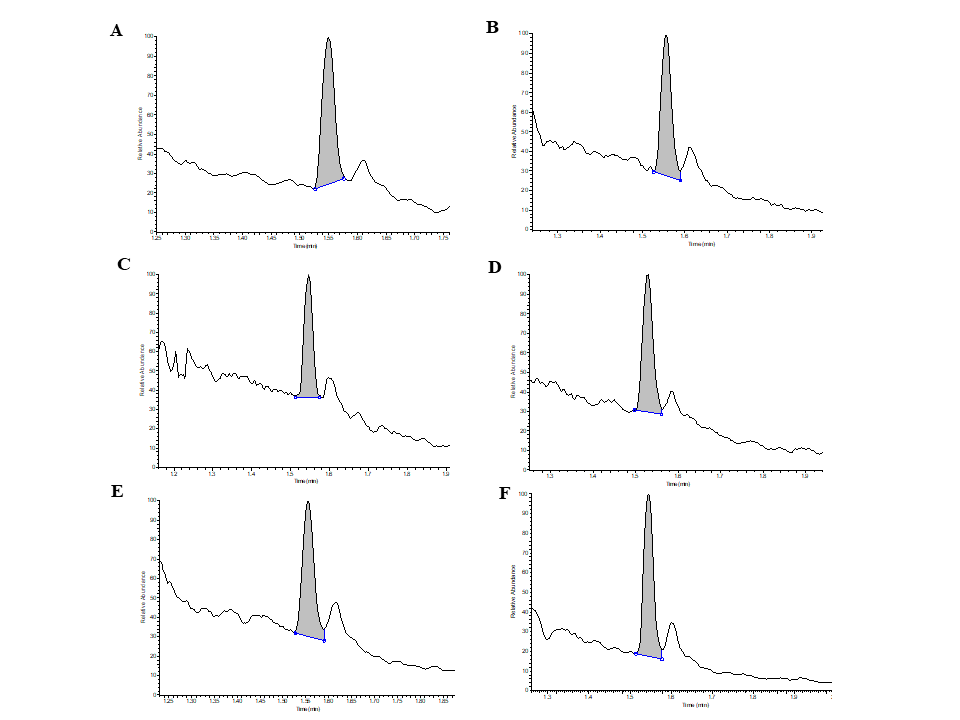


***Figure S7. Representative chromatograms for serum propionate in HFD-induced obesity in rats.*** *A: control, B: Dapa, C: HFD, D: HFD/Dapa, E: HFD/IF, F: HFD/Dapa+IF.* *The y-axis represents relative abundance and the x-axis represents time (min).*


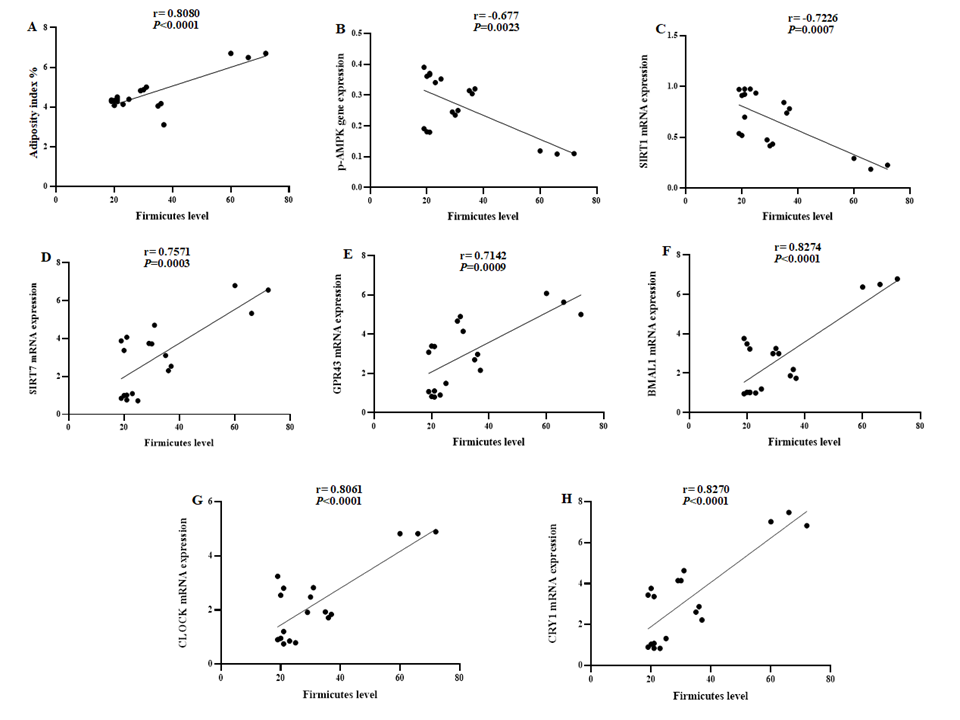


***Figure S8. Correlation between Firmicutes and adipose tissue parameters.*** *Correlation was done in all the studied groups collectively (total n = 18). The groups’ data were aligned vertically and the parameters horizontally. Each raw contained the parameters' values from the same rat.*


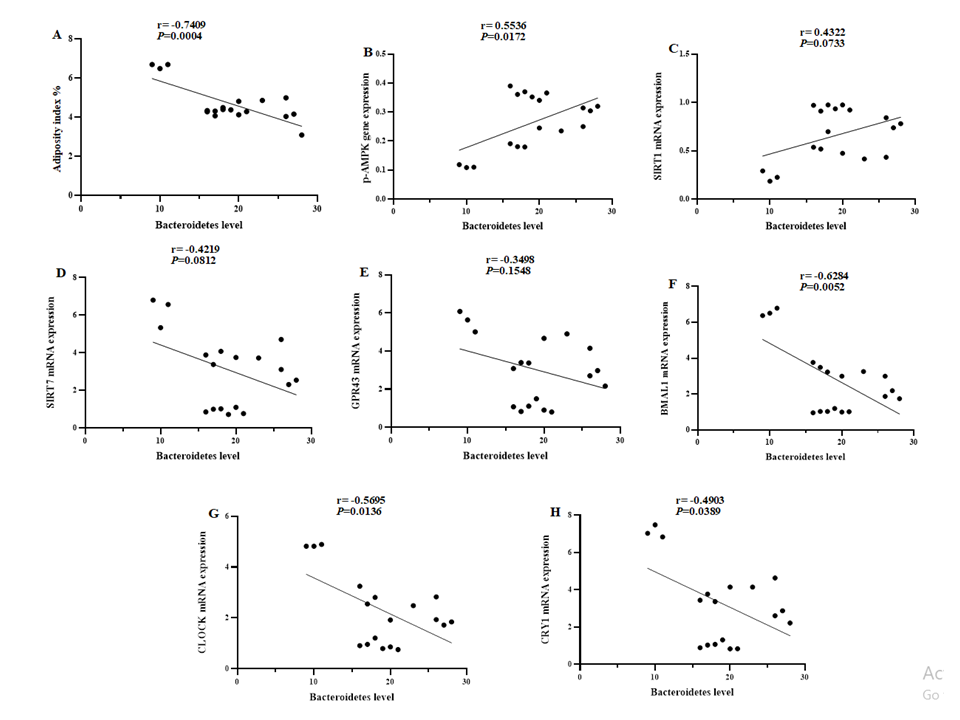


***Figure S9. Correlation between Bacteroidetes and adipose tissue parameters.*** *Correlation was done in all the studied groups collectively (total n = 18). The groups’ data were aligned vertically and the parameters horizontally. Each raw contained the parameters' values from the same rat.*
